# Revising the measurement of Eriksonian ego identity in contemporary Italian adolescents: the EPSI-IT

**DOI:** 10.3389/fpsyg.2026.1927380

**Published:** 2026-09-29

**Authors:** David Vagni, Antonio Tintori, Giulia Ciancimino, Leone G. Orvieto, Loredana Cerbara

**Affiliations:** 1 Institute for Research and Innovation in Biomedicine, National Research Council of Italy, Rome, Italy; 2 Institute for Research on Population and Social Policies, National Research Council of Italy, Rome, Italy; 3 Department of Education Science, University of Roma Tre, Rome, Italy

**Keywords:** bifactor model, Erikson Psychosocial Stage Inventory, gender identity, measurement invariance, social identity

## Abstract

**Introduction:**

The Identity subscale of the Erikson Psychosocial Stage Inventory (EPSI) is the most widely used brief measure of Eriksonian ego identity, but it carries a historically situated gender item and has no published Italian validation. Instruments inherit the assumptions of the era that produced them, and an item can lose validity as those assumptions shift.

**Methods:**

In two waves of an Italian school cohort in Rome (2025, *N* = 3,068; 2026, *N* = 2,896), we examined the original EPSI and developed the revised EPSI-IT.

**Results:**

Among the models compared, a bifactor solution fit best in both waves (EPSI-IT ω_H_ = 0.64, ECV = 0.54), though absolute fit was moderate; the structure supports a total score complemented by a synthesis subscale, whereas clarity (reverse-keyed confusion) adds little beyond the general factor. The 11-item EPSI-IT showed a coherent structure and a theory-consistent nomological network, and the harmonized core was invariant across groups and waves in terms of the loading level, with a minor intercept-level decrement. Across-wave criterion attenuation appeared equally on the constant harmonized items, arguing against an explanation specific to the revision. The gender item did not function as an ego-identity indicator and was removed.

**Discussion:**

We recommend the total score as the primary index, complemented by the synthesis subscale, and report preliminary grade-specific reference values for research use. More broadly, a historically situated item—here gender-related certainty used as a marker of ego identity—can come to function as separable content in a contemporary cohort.

## 1 Introduction

Erikson placed the formation of a coherent ego identity at the center of adolescent development: the young person integrates childhood identifications into a sense of self that is continuous over time and recognizable to others, and the failure to do so leaves them in role confusion (Erikson, 1950, 1968). Operationally, this coherence has two faces: a positively experienced synthesis and its negative pole, a felt confusion or lack of clarity about who one is. Adolescents work out identity across several domains at once, vocational, interpersonal, and ideological or values-based among them (Branje et al., 2021; Crocetti et al., 2010); the Identity subscale of the Erikson Psychosocial Stage Inventory (EPSI; Rosenthal et al., 1981) targets the global sense of coherence that cuts across these domains rather than any single one. A synthesized identity is a protective psychosocial resource, as a weak or diffuse identity is repeatedly linked to depressive symptoms, internalizing problems, and, net of depression, to suicidal ideation (Bogaerts et al., 2021; Branje et al., 2021; Sękowski et al., 2024). Identity work is also age-graded: it intensifies from early to middle adolescence, and identity synthesis follows a normative curve that dips over the early secondary-school years before consolidating later, with identity confusion moving in the opposite direction over the same span (Bogaerts et al., 2021; Branje et al., 2021). This coherence is not fixed once achieved but keeps being negotiated, and it now forms under social conditions Erikson did not anticipate, including the migration of much adolescent social life into digital environments. A measure of identity coherence that travels well across cohorts, therefore, matters for both developmental science and the applied assessment of psychosocial vulnerability.

The EPSI Identity subscale, the most widely used brief operationalization of Eriksonian identity, comprises 12 items split between positively keyed synthesis and reverse-keyed confusion content. Its dimensionality was debated for decades, since theory implies a single bipolar continuum while factor analyses recurrently extract two correlated factors (Gray et al., 1986), but the debate has converged: in emerging adults (Schwartz et al., 2009) and adolescents (Dimitrova et al., 2019; Hatano et al., 2018) a bifactor model, a general identity factor with synthesis and confusion specifics, outperforms one- and two-factor solutions, and the synthesis/confusion split is largely a keying method effect, as for the Rosenberg Self-Esteem Scale (Gnambs et al., 2018). In Italy the subscale has neither published norms nor reference values, and it reached only configural and partial metric equivalence in the US–Japan comparison of Dimitrova et al. (2019), so cut-offs borrowed from other countries cannot be adopted uncritically, the more so because Italian adolescents show comparatively prolonged identity moratoria, with more in-depth exploration and reconsideration than their northern-European and U. S. peers (Crocetti et al., 2010). A contemporary Italian identity measure exists (the AIDA; Musetti et al., 2021), but it targets identity diffusion within a clinical framework rather than the synthesis–confusion continuum captured by the EPSI Identity subscale.

A validated measure is not fixed for good: instruments carry the social assumptions of the era that built them, and an item can lose validity as those assumptions shift. The clearest precedent is gender-role-attitude measurement, where scales anchored in the 1970s and 1980s “male-breadwinner” model proved non-invariant across cohorts and were formally revised, because items that once discriminated a latent attitude came to register only assent to or rejection of an anachronistic stereotype (Constantin and Voicu, 2015; Seddig and Lomazzi, 2019; Walter, 2018a, 2018b). When an item’s meaning drifts in this way, scoring it as though stable conflates response shift with true change in the latent trait (Leitgöb et al., 2023). The EPSI inherited Erikson’s framing, in which sexual identity and gender roles belonged to ego identity (Erikson, 1968), and early operationalizations did the same: Marcia (1966) status paradigm surveyed sex-role and ideological domains as identity-relevant, and brief inventories, the EPSI among them, used the certainty of being a man or a woman as an indicator of global identity coherence. That anchoring is a candidate for exactly the drift documented in the gender-role literature.

Whether the EPSI’s gender item still indexes ego identity is thus an open empirical question rather than an assumption. The clinical identity-development tradition continues to treat subjective gender identity as integral to identity functioning, especially in adolescents (the AIDA; Goth et al., 2012; Musetti et al., 2021), whereas contemporary developmental work in general-population samples distinguishes gender identification from gender-role ideology and treats the two as separable constructs (Manago et al., 2022), against a backdrop in which gender identity has grown more salient and articulated in recent cohorts (Peruzzi et al., 2022). If certainty about what it means to be a man or a woman once proxied global self-coherence in the Eriksonian framing the EPSI inherited, it need not do so for an adolescent in 2026. According to contemporary process theories of identity development, in which commitments are formed, explored in depth, and reconsidered across adolescence (Branje et al., 2021; Crocetti et al., 2010), disagreement is more readily read as reconsideration of commitment, a normative dimension of the process, than as identity diffusion, which is the absence of commitment altogether. The distinction matters particularly in this population, whose adolescents, as noted above, reconsider more rather than less than their Northern-European and U. S. peers (Crocetti et al., 2010).

This article assesses the structure and validity of the EPSI Identity scale in Italian adolescents, provides preliminary reference values, and tests what happens when a historically situated item is read by a contemporary cohort. We use a two-wave cohort of Italian early adolescents in which the Italian wording of the EPSI Identity subscale was deliberately revised between waves (the gender item removed, three items reworded, one replaced). The two waves function largely as two studies, differing in both instrument and sample: the original EPSI and the revised form are not the same item set, and the cohort turns over between waves (a 1,963-respondent panel measured twice, plus 1,105 respondents only in Study 1 and 933 only in Study 2). To ensure the overlapping panel does not drive the conclusions, we verified each study’s central finding in its non-overlapping subsample: the gender-item anomaly among the Study-1-only leavers, and the EPSI-IT bifactor structure among the Study-2-only entrants. Because the EPSI reached only partial cross-national equivalence (Dimitrova et al., 2019), we label the revised form the EPSI-IT, an Italian adaptation rather than a universal successor. We validate it against a nomological network organized around three expectations. Convergent criteria (self-esteem, life satisfaction, future outlook) should relate positively, because a synthesized identity supplies the stable self-evaluation and the sense of temporal continuity on which positive self-regard and forward engagement rest (Branje et al., 2021; Erikson, 1968). Internalizing criteria (psychological distress, loneliness, alexithymia, body-image concern) should relate inversely, and more strongly to the confusion pole than to synthesis, since it is the felt incoherence about who one is, rather than the absence of commitments, that co-occurs with dysregulated affect and with the depressive symptoms repeatedly tied to identity diffusion (Bogaerts et al., 2021; Sękowski et al., 2024). A block of ideological and values measures (gender-stereotype endorsement, political orientation, religiosity, meritocratic values) should be largely unrelated: identity coherence concerns the form of the self-concept rather than its ideological content, so substantial associations here would indicate construct-irrelevant variance rather than validity. Study 1 characterizes the factor structure of the original EPSI in Wave 1 and isolates the behavior of the gender item, its place in the general factor, its differential functioning, and its relations to ego identity; Study 2 develops and validates the EPSI-IT in Wave 2: structure, item performance, reliability, measurement invariance across waves, gender, selection groups, and school track, the nomological network (reported wave by wave, with a decomposition that tests whether any across-wave change is specific to the revision), gender differences at the item level, and preliminary reference values.

## 2 Materials and methods

### 2.1 Participants and procedure

Data come from a longitudinal study of Italian adolescents [project name and coordinating institution masked for peer review]. Public upper-secondary schools in the municipality of Rome (Roma Capitale) were sampled through a cluster design stratified on educational track [academic *liceo* (lyceum), technical, and vocational] and the educational level of the school’s catchment area [the 155 urban zones (zone urbanistiche) of Roma Capitale grouped into high, medium, and low tertiary-education areas, 2021 census]. This yielded 25 schools. The cohort is followed from the first to the fifth year of upper-secondary school. The questionnaire was self-administered on a computer: respondents read the items and entered their own responses, for privacy, with trained staff present in the room to answer clarification requests. Participation was with parental consent and approval from the coordinating institution’s research ethics committee (masked for peer review).

The present analyses use the first two waves. In Wave 1 (2025), the cohort was in the first year (*M* age = 14.3, *SD* = 0.56; *n* = 3,068 with valid identity responses); in Wave 2 (2026), the same cohort was in the second year (*n* = 2,896). Item-level missingness on the identity scale was low (≤ 2% per item); ordinal CFA used complete cases (listwise deletion) under WLSMV, giving analytic samples of *n* = 3,010 (Wave 1) and *n* = 2,768 (Wave 2). Records were linked across waves by student code after a deduplication procedure; 1,963 respondents formed the reliable two-wave linked panel, of whom 1,899 with complete harmonized-scale data in both waves entered the within-person change analyses (Figure 1). Because the cohort advances as a school population, each wave also contains wave-unique respondents: leavers present only in 2025 (*n* = 1,105), and entrants present only in 2026 (*n* = 933). Entrants were slightly older for grade (mean age-for-grade +0.47 vs. +0.28 in the panel, age-for-grade being age minus the modal age for the grade, 14 in year one and 15 in year two), and leavers reported somewhat lower achievement, so selection groups are modeled explicitly in the invariance analyses. Gender was self-reported as female (≈ 43%), male (≈ 56%), or a third “other” option (*n* = 23 in each wave, 0.7–0.8%); the male majority reflects the track-stratified oversampling of technical and vocational schools. Given the rising salience of non-binary identities in recent cohorts, we report the “other” category explicitly rather than folding it away. It is too small for separate modeling, so gender-group comparisons contrast girls and boys; the multigroup CFAs exclude respondents reporting “other” or with missing gender, who are retained in the full-sample models. Inferences about non-binary adolescents are correspondingly limited. The *n* = 23 “other” count refers to the assessed samples (20 and 19 in the listwise analytic samples). Missing gender in Wave 2 was confined to entrants (75 of the 933 assessed; 73 in the analytic sample): the gender field was not mandatory in Wave 2, so a few new respondents left it blank, whereas panel members’ gender was carried forward from Wave 1 (which had none missing).

**Figure 1:**
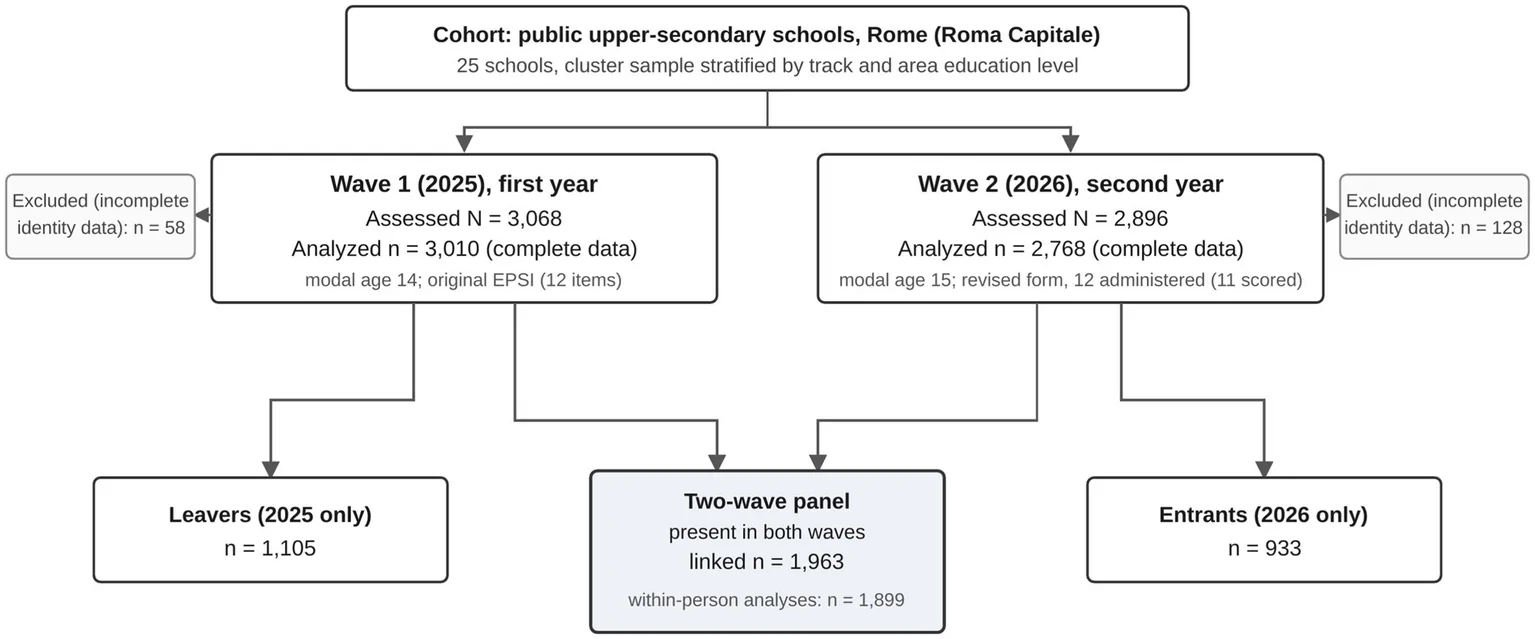
Participant flow and two-wave panel structure, showing the overlapping panel and the wave-unique leavers (Study 1 only) and entrants (Study 2 only). Panel members are linked across waves by student code; panel, leavers, and entrants partition each wave’s assessed sample. Gender (full sample): female ≈ 43%, male ≈ 56%, other ≈ 0.8% (*n* = 23 per wave). The 8 wave-common items form the harmonized score used for the invariance and change analyses; within-person change analyses use the panel members’ complete responses on those items in both waves (*n* = 1,899).

### 2.2 Measures

#### 2.2.1 Ego identity

The 12-item Identity subscale of the Erikson Psychosocial Stage Inventory (EPSI; Rosenthal et al., 1981), rated 1–5, with positively keyed *synthesis* items and reverse-keyed *confusion* items. Throughout, the confusion items are recoded so that higher scores indicate greater coherence, and we label that pole *clarity*; all reported scores are coherence-keyed (higher = stronger ego identity).

#### 2.2.2 Item adaptation and provenance

No published, validated Italian version of the EPSI Identity subscale exists. The subscale had appeared in unpublished Italian research with non-equivalent wordings (Picariello, 2015; Iadanza, 2022), and the Wave-1 Italian items used here, a working adaptation prepared by the research team, match neither verbatim. One adaptation decision bears directly on the gender item: the 1981 English “what it means to be female/male” was rendered as “cosa significhi essere donna/uomo” (woman/man), not the literal “femmina/maschio.” In Italian, “femmina/maschio” denotes biological sex; applied to a psychological self-description, it reads as crude and would likely be construed by first-year adolescents as a question about human biology. The item, as administered, therefore, concerns the social meaning of being a woman or a man, in line with the distinction between gender and sex that was not yet clearly drawn when the EPSI was written. We did not conduct a formal forward- and back-translation or expert-panel reconciliation; we note this against the ITC Guidelines for Translating and Adapting Tests (International Test Commission, 2018) and the Standards for Educational and Psychological Testing (American Educational Research Association and American Psychological Association and National Council on Measurement in Education, 2014), and the absence of a validated Italian form is itself part of the rationale for developing the EPSI-IT. Each wave was collected between January and March of the wave year. Exploratory analyses of the Wave-1 data, run before the Wave-2 questionnaire was finalized, flagged the anomalous gender item and informed the Wave-2 revision. The Study-1 analyses below therefore document the empirical basis of that decision on the same Wave-1 data, while the Wave-2 analyses evaluate the revised form prospectively. Neither stage was preregistered.

#### 2.2.3 The field revision (EPSI to EPSI-IT)

Each item was classified by the magnitude of change (Supplementary Table S1): 4 unchanged (items 3, 6, 7, 10), 4 lightly reworded and treated as equivalent (items 1, 2, 4, 11), 3 substantively reworded, and 1 replaced. The substantive rewordings were motivated on content grounds. Item 5 (“I have everything under control” became “I feel able to manage what happens to me”) because the original reads as near-obsessive control rather than healthy coherence; item 9 (“I like myself and am proud of what I believe in” became “I like who I am”) because pride in one’s beliefs is ideologically loaded (for example, political or religious) and, with item 5, indexed an inflexibility at odds with a contemporary view of adaptive identity; item 12 (“I do not feel very involved in what I do” became “I do not think about building my future”) because the original may capture ordinary boredom, and the new wording, with items 2 and 7, was intended to probe a future-orientation dimension. Future orientation is a plausible facet of Eriksonian identity, which entails a self-experienced as continuous into an anticipated future (Erikson, 1968); as reported in the Results, this dimension did not hold up. The gender item (8, “I have a strong sense of what it means to be a woman/man”) was removed for the reasons set out above. It was provisionally replaced by a generic stability item (14, “I do not see why I should change”), included to adjudicate between two readings the authors had debated: as a marker of synthesis (a settled sense of self) or, conversely, as rigidity, the lack of the flexibility that a contemporary view of adaptive identity values (cf. items 5 and 9). It behaved as neither, carrying little general-factor variance, and was dropped (see Results). This yields three scorable forms:

- *EPSI (original):* the 12 historically worded items (Wave 1).
- *EPSI-IT:* the revised 12-item form (Wave 2); the 11-item version omits the weak replacement item (see Results).
- *Harmonized 8-item form:* the 8 items common and equivalent across waves (the 4 unchanged plus the 4 lightly reworded: items 1, 2, 3, 4, 6, 7, 10, 11). This is the only fully wave-comparable score and is used for all longitudinal and cross-wave invariance analyses.

#### 2.2.4 Criterion and correlate measures

Self-esteem (RSES; Rosenberg, 1965; Italian validation, Prezza et al., 1997), psychological distress (K10; Kessler et al., 2002; Italian validation, Carrà et al., 2011), alexithymia (six-item PAQ-S; Preece et al., 2023; administered in an Italian working translation, with no published Italian validation available, but with adequate internal consistency in the present sample, ω = 0.77 and 0.79 in 2025 and 2026), aggression (BPAQ; Buss and Perry, 1992; Italian validation, Fossati et al., 2003), body-image concern, single 0–10 items for life satisfaction, loneliness, and future outlook, social-isolation, hikikomori, and hyperconnection indicators, bullying and cyber-victimization, cyber-perpetration, substance exposure and tolerance, violence tolerance, trust in others, school belonging, negative school climate, academic achievement (self-reported, three categories: low, medium, high), and, as a discriminant block, gender-stereotype endorsement (GAB-A; Tintori et al., 2026: the GSAS, GRAS, and GTI subscales), political orientation, religiosity, and meritocratic values. Each study-developed criterion was a brief self-report indicator (single item or short scale): the body-image-concern measure; the single 0–10 items (life satisfaction, loneliness, future outlook); the social-isolation, hikikomori, and hyperconnection indicators; bullying and cyber-victimization and cyber-perpetration; substance exposure and tolerance; violence tolerance; trust in others, school belonging, and negative school climate; and the meritocratic-value indicator. Trust in others and school belonging are coherence-coded (higher = more); social isolation, hyperconnection, hikikomori, and the top-box meritocratic value are binary. The item-by-item mapping (2025 wording, 2026 wording, change category, harmonized-set membership) is given in Supplementary Table S1, and the administration-ready 11-item form, in Italian with English glosses, in Supplementary Appendix 1 (Open Materials). The original English EPSI items are third-party copyrighted material and are not reproduced; they are available in Rosenthal et al. (1981). The recommended 11-item EPSI-IT comprises provenance items 1, 2, 3, 4, 6, 7, 10, 11, 13, 15, 16 (synthesis: 2, 4, 6, 13, 15; clarity: 1, 3, 7, 10, 11, 16), renumbered 1–11 for administration.

### 2.3 Analytic strategy

Factor models used ordinal WLSMV (theta parameterization). For each form we compared a unidimensional model, a correlated two-factor model (synthesis and clarity, coherence-keyed), and a bifactor model (general identity factor plus synthesis and clarity specifics), reporting scaled χ^2^, *df*, CFI, TLI, RMSEA with 90% CI, and SRMR, plus bifactor indices [ECV, PUC, ω_H_, ω_HS_, H (construct replicability), and factor determinacy; Rodriguez et al., 2016a, 2016b; Reise, 2012], computed from the confirmatory bifactor solution with the same formulas for both forms. Item-level standardized loadings are reported from the correlated two-factor model (Supplementary Table S2). Although the bifactor fit best on the omnibus indices, the clarity specific factor was weakly defined for the EPSI-IT (negligible specific reliability), so we relied on the sign-consistent two-factor solution both for item-level interpretation and for the measurement-invariance tests, a multigroup bifactor with a near-empty clarity specific being weakly identified across groups. Because the total score is a composite of the same items, item-level invariance in this representation is the relevant practical evidence for comparing total scores, though it does not by itself establish invariance of the bifactor general factor. An exploratory structure was used, polychoric EFA (weighted least squares extraction, oblimin rotation), with the number of factors guided by parallel analysis (Horn, 1965; 500 simulated datasets). The confirmatory comparison of unidimensional, correlated two-factor, and bifactor models is the primary test of dimensionality; the EFA served a diagnostic role, characterizing item behavior (notably the anomalous gender item), and no item was retained or removed on the basis of the EFA. The gender-item revision was, moreover, decided from exploratory Wave-1 analyses before the Wave-2 instrument was finalized (see Measures), so Study 1 documents the empirical basis of a decision that was prospective with respect to Wave 2, rather than a search for items to drop. The EPSI-IT structure was cross-validated on a random split (EFA on one half, confirmatory bifactor on the held-out half), and a third (future-orientation) factor was tested both as a correlated factor and as a three-specific bifactor. Measurement invariance of the harmonized 8-item form was tested across waves, gender, selection groups, and school track with ordinal Wu and Estabrook (2016) identification (theta parameterization), in a four-step nested sequence (configural; equal thresholds; equal thresholds and loadings, the metric level; equal thresholds, loadings, and item intercepts, the scalar level), separating threshold from loading invariance as the ordinal parameterization requires. Change was evaluated between adjacent models in CFI, RMSEA, and SRMR (the ΔCFI ≤ 0.010 convention; Chen, 2007; Cheung and Rensvold, 2002; Putnick and Bornstein, 2016), reading RMSEA and SRMR as more stable than scaled ΔCFI under added categorical constraints (Xia and Yang, 2019; Sass et al., 2014). Because the waves share a large overlapping panel, the cross-wave contrast was estimated primarily as longitudinal invariance within the panel (a single-group model with both occasions and correlated residuals for repeated items), with the between-cohort multigroup comparison retained as a secondary, descriptive analysis; latent means were estimated in SD units (reference occasion or group fixed at 0). Gender and school-track invariance of the recommended 11-item EPSI-IT were tested within Wave 2 on the same four-step sequence, to match the form used for the reference values. As a complement, the 11-item form was also submitted to multiple-group alignment (Asparouhov and Muthén, 2014), estimated per factor on group-specific loadings and intercepts with items treated as continuous (sirt 4.2–133; Robitzsch, 2025), which quantifies approximate invariance item by item without imposing exact constraints; because alignment is designed for many groups, it is reported as a robustness check on the confirmatory sequence. Reliability used McDonald’s ω (total, hierarchical, and per subscale; McDonald, 1999), with bootstrap 95% confidence intervals for the subscale coefficients. Item-8 differential functioning by gender was tested both as uniform DIF (a MIMIC direct effect within the bifactor) and as non-uniform DIF (a gender difference in the item’s loading). The nomological network was estimated separately by wave (Wave 1 on the original EPSI, Wave 2 on the EPSI-IT) and, in parallel, on the harmonized 8-item form in both waves. Because the harmonized items are common across waves (four unchanged and four lightly reworded), the best available cross-wave anchor rather than a perfect constant-instrument control, we used them to decompose any across-wave change in criterion validity into a wave (period) and a scale-revision component, regressing the full-scale attenuation on the harmonized-only attenuation. Criterion correlations were Pearson throughout, with Benjamini–Hochberg control of the false discovery rate across the nomological coefficients (Benjamini and Hochberg, 1995). Four criteria are binary (social isolation, hyperconnection, hikikomori, and the top-box meritocratic value), so their coefficients are point-biserial correlations; these are attenuated by the dichotomous split, most strongly for the rare hikikomori category (≈ 6–7% prevalence). Gender differences were quantified at the scale and item level (Cohen’s *d*), and we report the associations of EPSI-IT scores with age and academic achievement. To characterize the temporal ordering of the identity–distress association, we fitted a cross-lagged panel model on the linked panel (harmonized identity score and K10 at both waves, MLR estimation with full-information maximum likelihood for missing data, autoregressive and cross-lagged paths with within-wave residual correlations freely estimated); it is reported as a descriptive adjunct to the gender-gap analysis rather than as a test of causal precedence. The two studies map onto the two forms: Study 1 characterizes the original EPSI in Wave 1 and the gender item; Study 2 develops and validates the EPSI-IT in Wave 2 and reports invariance, change, the nomological network, gender, individual-difference correlates, and reference values. Analyses were run in R 4.6.0 (R Core Team, 2026) with lavaan 0.6–21 (Rosseel, 2012) for ordinal CFA and invariance, semTools 0.5–8 (Jorgensen et al., 2022) for the Wu–Estabrook syntax, and psych 2.6.5 (Revelle, 2025) for polychoric EFA and parallel analysis.

## 3 Results

### 3.1 Study 1: structure of the original EPSI and the gender item (wave 1)

Fit for the three competing models is in Table 1. The unidimensional model fit poorly, χ^2^(54) = 4599.4, CFI = 0.775, TLI = 0.726, RMSEA = 0.167 [0.163, 0.171], SRMR = 0.119; the correlated two-factor model improved fit, χ^2^(53) = 1949.0, CFI = 0.906, RMSEA = 0.109 [0.105, 0.113], SRMR = 0.065; and the bifactor model fit best, χ^2^(42) = 1418.9, CFI = 0.932, TLI = 0.893, RMSEA = 0.104 [0.100, 0.109], SRMR = 0.048, WRMR = 2.54, replicating prior EPSI work (Schwartz et al., 2009; Dimitrova et al., 2019). Model choice here is comparative: the bifactor is preferred over the unidimensional and two-factor models on the same indices, and its absolute values remain suboptimal and are interpreted cautiously. Conventional cutoffs derived under normal-theory maximum likelihood with continuous data do not transfer directly to ordinal WLSMV estimation, and categorical estimators may in fact yield optimistic rather than conservative CFI and TLI values (Xia and Yang, 2019); absolute and comparative indices are therefore read jointly, with brief, low-degrees-of-freedom models in large samples adding further imprecision (Kenny et al., 2015). General-factor saturation was appreciable (ECV = 0.564, ω = 0.859, ω_H_ = 0.621, PUC = 0.545), supporting total-score interpretation (Rodriguez et al., 2016a); the two specifics were asymmetric, carrying reliable variance beyond the general factor for synthesis (ω_HS_ = 0.57) but little for confusion (ω_HS_ = 0.12).

**Table 1:** Factor model fit (ordinal WLSMV).

Form/model	*N*	χ^2^	df	CFI	TLI	RMSEA [90% CI]	SRMR
EPSI original (2025), 12 items	3,010						
Unidimensional		4599.4	54	0.775	0.726	0.167 [0.163, 0.171]	0.119
Two-factor (synthesis/clarity)		1949.0	53	0.906	0.883	0.109 [0.105, 0.113]	0.065
Bifactor (g + 2 specifics)		1418.9	42	**0.932**	0.893	0.104 [0.100, 0.109]	0.048
EPSI-IT (2026), 11 items	2,768						
Unidimensional		4136.4	44	0.732	0.665	0.183 [0.179, 0.188]	0.134
Two-factor (synthesis/clarity)		1719.0	43	0.890	0.860	0.119 [0.114, 0.124]	0.067
Three-factor (+ future)		2453.6	41	0.842	0.788	0.146 [0.141, 0.151]	0.087
Bifactor (g + 2 specifics)		1236.5	33	**0.921**	0.869	0.115 [0.109, 0.120]	0.053
Harmonized 8-item (same items, by wave)							
Two-factor, 2025	3,010	1040.3	19	0.905	0.861	0.134 [0.127, 0.141]	0.063
Two-factor, 2026	2,768	890.5	19	0.910	0.868	0.129 [0.122, 0.136]	0.060

Ordinal WLSMV (theta parameterization); robust scaled fit statistics, all χ^2^ *p* < 0.001. N = listwise analysis sample. CFI, comparative fit index; TLI, Tucker–Lewis index; RMSEA, root mean square error of approximation (90% CI in brackets); SRMR, standardized root mean square residual. The harmonized 8-item form comprises the items common to both waves and is reported separately by wave; the EPSI-IT is the recommended 11-item form (item 14 omitted, see Results). Bold values indicate the selected (best-fitting) model.

The gender item (item 8) was anomalous. In the bifactor model, its standardized loading on the general identity factor was −0.02 (all other items 0.31 to 0.76) and its communality the lowest in the set (0.15); in the correlated two-factor model, it was likewise the weakest synthesis indicator by a wide margin (0.23 vs. 0.64–0.74) (Supplementary Table S2). This was not a floor effect (largest *SD* = 1.41, well-spread distribution). In polychoric EFA, the item did not drop out but loaded essentially alone, as a singlet that no other item populated (loading 0.89, communality 0.81; parallel analysis indicated four factors); a one-item factor is not a validated dimension, but it confirms the item does not cohere with the ego-identity content. A MIMIC test within the bifactor model found no uniform gender DIF [gender-to-item direct effect = 0.06, *p* = 0.20; LRT Δχ^2^(1) = 1.55, *p* = 0.21], and freeing the item’s loading across gender showed no non-uniform DIF either [Δχ^2^(1) = 2.3, *p* = 0.13; standardized loadings 0.19 in girls, 0.26 in boys]; an apparent DIF under the misspecified unidimensional model did not survive correct specification. The item’s relations to gender-stereotype, political, religious, and meritocratic measures were near zero (|*r*| ≤ 0.08); the ordinary identity items related to stereotype endorsement more (0.15–0.17) than the gender item did (0.01). It’s one systematic external signal was gender-polarized: its correlation with stereotype endorsement reversed sign across gender (girls *r* = −0.09, boys *r* = +0.05; *z* = −3.79, *p* < 0.001), whereas its relation to the rest of the identity scale did not differ by gender (*z* = −1.52, *p* = 0.13). The anomaly was not an artifact of the panel shared with Study 2: in the Study-1-only leavers (*n* = 1,067) the item again loaded weakly on the general factor (−0.11), with the bifactor model fitting as in the full sample (CFI = 0.935) (Supplementary Table S3).

### 3.2 Study 2: development and validation of the EPSI-IT (wave 2)

With the gender item removed by design, no 2026 item carried gender-related content. Parallel analysis indicated three factors. Item review of the full 12-item form flagged a single weak item. The replacement item 14 loaded near zero on the general factor (−0.07), with the lowest communality (0.27) and item-rest correlation (0.18), and removing it improved hierarchical reliability. Every other item met retention criteria, and the common items were stable across waves (item-rest 2025/2026, e.g., 0.58/0.54 and 0.51/0.47). We therefore adopted the 11-item EPSI-IT (item 14 omitted), reported as a transparent record of a constructive replacement that did not perform. On this form, competing structural models were compared (Table 1). The bifactor model fit best, χ^2^(33) = 1236.5, CFI = 0.921, TLI = 0.869, RMSEA = 0.115 [0.109, 0.120], SRMR = 0.053, WRMR = 2.61, ahead of the two-factor (CFI = 0.890) and unidimensional (CFI = 0.732) models. As in Study 1, the bifactor is selected on comparative grounds, and its absolute indices are read with the same caveat about categorical estimation. This structure replicated on the random split (confirmatory bifactor on the held-out half, CFI = 0.888, RMSEA = 0.125, absolute fit modest) and held in the Study-2-only entrants, the respondents not shared with Study 1 (*n* = 850, bifactor CFI = 0.921, RMSEA = 0.115), with full scalar invariance between entrants and the panel (Supplementary Table S4). General-factor saturation was appreciable (ω_H_ = 0.64; see Reliability), and the two specifics reproduced synthesis and clarity, though only the synthesis specific carried reliable variance beyond the general factor. Item distributions were adequate (no floor or ceiling, all response categories used by more than 5% of respondents, no rescoring indicated; two items showed mild bimodality). Bifactor indices and the resulting scoring recommendation are reported below.

A three-factor solution adding a future-orientation factor (items 2, 7, 16) fit better in EFA but not in confirmatory modeling: the simple-structure three-factor model fit worse than two factors, χ^2^(41) = 2453.6, CFI = 0.842, RMSEA = 0.146 [0.141, 0.151], against the two-factor χ^2^(43) = 1719.0, CFI = 0.890, RMSEA = 0.119 [0.114, 0.124]; the future factor was unreliable (α = 0.61) and not separable (factor correlations 0.69 to 0.76). A bifactor adding a third, future-orientation specific (alongside the general factor) fit worse still, χ^2^(33) = 2434.7, CFI = 0.843, RMSEA = 0.162, and produced an incoherent specific factor (mixed-sign loadings; item 16 = −0.54). Future orientation is therefore not a separable dimension in these items. Reinserting item 14 into a future factor did not help (α = 0.44); in EFA, item 14 loaded on synthesis (0.60), not future.

### 3.3 Measurement invariance and 1-year change (harmonized 8-item form)

On the eight common items, a two-factor synthesis/clarity model was tested for measurement invariance under ordinal Wu–Estabrook identification, in a four-step sequence (configural, then equal thresholds, then thresholds plus loadings, then thresholds, loadings, and intercepts), evaluating change between adjacent nested models (Chen, 2007; Table 2). For the cross-wave question, which is the central one in a panel, the primary test was longitudinal invariance within the panel itself (the same 1,899 respondents measured in both waves, with correlated residuals for repeated items); this avoids treating the overlapping 2025 and 2026 samples as independent groups, which a between-cohort multigroup comparison would do. In the longitudinal panel, threshold invariance held (ΔCFI = −0.006) and loading invariance held (ΔCFI = +0.004); the intercept step showed a small decrement (ΔCFI = −0.019) whose largest residual involved item 1, one of the four lightly reworded common items, and the latent synthesis change was unaffected by it. Scalar (intercept) invariance also held across gender, selection groups, and school track (configural-to-intercept ΔCFI = +0.002, +0.009, +0.011). At the threshold step, the scaled CFI dipped by about 0.013–0.015 before recovering once loadings were added; this is the known behavior of scaled fit statistics under added constraints with categorical data, and ΔRMSEA and ΔSRMR, which are more stable in this setting, supported invariance at every step (Xia and Yang, 2019). Because leavers contributed only Wave-1 and entrants only Wave-2 responses while the panel spans both, the selection grouping is partly confounded with wave and is not a pure selection contrast. Absolute fit was modest, as expected for a brief, low-degrees-of-freedom ordinal model estimated by WLSMV from polychoric correlations in a large sample, a setting in which RMSEA and CFI computed on the categorical metric are not comparable to the conventional continuous-data cutoffs (configural RMSEA ≈ 0.13; Kenny et al., 2015; Xia and Yang, 2019). Because the common items are unchanged or only lightly reworded across waves, they provide the best available cross-wave anchor rather than a perfect constant-instrument control. On this anchor, fit by wave was essentially equivalent (2025 CFI = 0.905, RMSEA = 0.134; 2026 CFI = 0.910, RMSEA = 0.129), with stable or slightly higher loadings in 2026 (synthesis Δ + 0.02 to +0.06; clarity Δ − 0.04 to +0.06) (Supplementary Table S2C) and a slight downward edge in hierarchical reliability (ω_H_ from 0.46 to 0.43). Where the 2026 form shows marginally lower reliability or criterion correlations than the 2025 original, this anchor comparison locates the drift in the wave (period and age) and in the removal of content-overlapping items, not in reduced validity of the EPSI-IT. The primary evidence on 1-year change is the within-person panel (*n* = 1,899 with complete harmonized data in both waves): synthesis declined slightly (*d_z_* = −0.116, 1-year retest *r* = 0.42) and clarity was stable (*d_z_* = +0.007, *r* = 0.50). Both the longitudinal latent-mean estimate (synthesis −0.169 SD, *z* = −5.92, *p* < 0.001; clarity −0.008 SD, *p* = 0.75) and the between-cohort comparison (synthesis −0.117 SD, *z* = −3.86, *p* < 0.001; clarity +0.006 SD, *p* = 0.83) agree in direction and significance, so the synthesis decline is not an artifact of sample composition, and clarity is best read as stable.

**Table 2:** Measurement invariance of the harmonized 8-item form (two-factor; ordinal WLSMV, Wu–Estabrook identification, four-step sequence).

Grouping	Level	CFI	RMSEA	SRMR	WRMR	ΔCFI	ΔRMSEA	ΔSRMR
Across waves: longitudinal panel (*n* = 1,899)	Configural	0.909	0.090	0.054	2.40	–	–	–
	Threshold	0.903	0.085	0.054	2.40	−0.006	−0.005	0.000
	Loading	0.907	0.081	0.055	2.44	+0.004	−0.004	0.000
	Intercept	0.888	0.087	0.054	2.74	**−0.019**	+0.006	0.000
Gender (F vs. M)	Configural	0.899	0.131	0.063	4.54	–	–	–
	Threshold	0.886	0.117	0.063	4.57	−0.013	−0.014	0.000
	Loading	0.899	0.105	0.063	4.65	+0.013	−0.012	0.000
	Intercept	0.901	0.099	0.063	4.98	+0.002	−0.006	0.000
Selection (stable/leaver/entrant)	Configural	0.904	0.133	0.063	4.53	–	–	–
	Threshold	0.890	0.114	0.063	4.57	−0.014	−0.019	0.000
	Loading	0.907	0.098	0.063	4.63	+0.017	−0.016	0.000
	Intercept	0.913	0.090	0.063	4.92	+0.006	−0.008	0.000
School track (lyceum/technical/vocational)	Configural	0.905	0.133	0.063	4.58	–	–	–
	Threshold	0.890	0.115	0.063	4.62	−0.015	−0.019	0.000
	Loading	0.907	0.099	0.064	4.67	+0.017	−0.016	0.000
	Intercept	0.916	0.089	0.064	4.88	+0.009	−0.010	0.000

Ordinal WLSMV (theta) under Wu and Estabrook (2016) identification. Per grouping, a four-step nested sequence: configural; equal thresholds; equal thresholds and loadings (the metric level); equal thresholds, loadings, and item intercepts (the scalar level). Δ columns are changes between adjacent nested models (Chen, 2007). CFI, RMSEA, and SRMR as in Table 1; WRMR, weighted root mean square residual, reported for completeness only and not used as a decision criterion (DiStefano et al., 2018). – = not applicable. Bold value indicates a ΔCFI exceeding the 0.010 cutoff.

### 3.4 Reliability, convergent and discriminant validity, and criterion relations

The total score was reliable (ω = 0.85) with appreciable general-factor saturation (ω_H_ = 0.64, ECV = 0.54, PUC = 0.55, *H* = 0.83, factor determinacy = 0.92), supporting its interpretation as a general identity index. The two specific factors were asymmetric: synthesis retained reliable variance beyond the general factor (ω_HS_ = 0.65, *H* = 0.79), whereas clarity did not (ω_HS_ = 0.01), its items behaving essentially as general-factor indicators. As standalone subscale scores both were internally consistent [synthesis ω = 0.79, 95% CI (0.77, 0.80); clarity ω = 0.76, (0.75, 0.78)]. We therefore recommend the total score as the primary index, complemented by the synthesis subscale; the clarity score, computed in forming the total, can be reported but should not be read as a dimension distinct from general identity.

The nomological network (full coefficients by wave in Supplementary Tables S5–S6) was theory-consistent, and we read it against what a measure of identity coherence should show. Convergent validity was clearest with self-esteem (*r* = 0.65) and, more moderately, with life satisfaction (0.49) and future outlook (0.51): a synthesized identity anchors a positive self-evaluation and a sense of forward engagement, the pattern expected if the scale indexes achieved coherence rather than transient mood (Branje et al., 2021). On the negative side, the total related inversely to psychological distress (−0.47), loneliness (−0.45), alexithymia (−0.42), and body-image concern (−0.35), reproducing the well-established link between weak or diffuse identity and internalizing difficulty (Bogaerts et al., 2021; Sękowski et al., 2024). This internalizing pole is diagnostic about the two facets: it was carried by clarity at least as much as by synthesis (distress −0.49 vs. −0.28), whereas synthesis was distinctively stronger only on future outlook, so the lack-of-clarity facet is the one entangled with distress, consistent with its confusion-keyed content. Discriminant validity was equally clear. Aggression related only moderately and inversely (total *r* = −0.34 and −0.32 across waves), and that relation too ran through clarity, matching an affective-dysregulation reading rather than a broad externalizing one; the remaining externalizing indicators (cyber-perpetration, substance use, violence tolerance) and the whole ideological and values block (gender-stereotype, political, religious, and meritocratic measures, |*r*| ≤ 0.17) were near zero, so the scale tracks identity coherence and not ideology or conduct. Four discriminant criteria are binary, so their point-biserial coefficients are conservative. The network was largely the same in girls and boys (two relations, distress and life satisfaction, were somewhat stronger in girls), and the gender-polarized signal that the original gender item alone carried was absent from the EPSI-IT.

The network held its shape across waves. The mild, near-uniform 2025–2026 attenuation was reproduced on the constant harmonized items (Supplementary Table S6), which locates it in the wave rather than in the revision. Modeling this directly across the 26 criteria, the full-scale and harmonized-only attenuation profiles were almost perfectly aligned (*r* = 0.98), and regressing one on the other gave a slope of 1.04 and *R*^2^ = 0.96. The attenuation is thus already present on the constant anchor, arguing against an explanation specific to the EPSI-IT revision; age, period, and residual wording effects cannot be separated in this design.

### 3.5 Reference values

Because no normative data exist for the EPSI Identity subscale, we provide preliminary grade-specific reference values (*M*, *SD*, percentiles on the 1–5 item-mean metric) by gender and track (Supplementary Table S7). Boys reported higher identity coherence than girls, most clearly on clarity (3.55 vs. 3.18) and the total (3.48 vs. 3.18). To support this comparison on the form to which the reference values apply, we tested invariance of the 11-item EPSI-IT within Wave 2 (not only the harmonized core; Supplementary Table S8): gender invariance held through the loading (metric) level, whereas the intercept step exceeded our ΔCFI criterion (ΔCFI = −0.019, against the 0.010 convention; ΔRMSEA and ΔSRMR negligible), so full scalar invariance is not established. The boys–girls latent difference nonetheless persisted under the scalar model (synthesis +0.27, clarity +0.48 SD) and was reproduced by the alignment analysis, so the higher male values are unlikely to be an artifact of the residual intercept non-invariance, though they cannot be fully separated from it (its substantive interpretation, largely the gender difference in distress, is taken up in the Discussion). School-track invariance of the 11-item form held to the scalar level (configural-to-intercept ΔCFI = +0.012), and track differences in the reference values were negligible. As a complement to this four-step sequence, a multiple-group alignment analysis (Asparouhov and Muthén, 2014) of the same 11-item form agreed with it (Supplementary Table S8B): loadings and intercepts were highly invariant across both groupings (alignment *R*^2^ ≥ 0.995 and ≥ 0.999, respectively), the small residual non-invariance localized to the substantively reworded items (largest intercept deviations on items 16 and 15), and the aligned latent means reproduced the confirmatory pattern, a boys-higher gap carried by clarity (+0.67 SD) far more than synthesis (+0.15 SD) and negligible track differences (all |aligned means| < 0.09). Because alignment is designed for many groups, we read it as corroboration rather than an independent test. Because the track-stratified design oversampled male-skewed schools, we also report a gender-reweighted (50/50) reference total (EPSI-IT ≈ 3.33). Wave 2 indexes the second year of upper secondary (modal age 15); reference values for Wave 1 (first year, modal age 14) on the original EPSI are in Supplementary Table S9. Within Wave 2, EPSI-IT scores were essentially independent of age (synthesis *r* = 0.06, *p* = 0.001; clarity and total nonsignificant) and showed a small positive association with academic achievement (synthesis 0.10, clarity 0.12, total 0.14).

### 3.6 Gender differences

Boys scored higher than girls in both waves, with a larger gap in 2025 (total *d* = 0.69) than in 2026 (*d* = 0.43). Because the gap also narrows on the common harmonized items (0.67 to 0.41), part of this reduction is a wave and age effect rather than a consequence of the revision. Item-level analysis (Supplementary Table S10) shows the gap is carried by the clarity items. Item 3 is the strongest indicator in both waves (*d* = 0.64 and 0.53), followed by items 1, 11, and 10; synthesis contributes far less. Two findings are relevant here. First, the gender item (8) showed the smallest gap of all in 2025 (*d* = 0.09): boys and girls reported near-equal certainty about their gender even as they differed on the ordinary identity items, consistent with item 8 indexing a distinct dimension. Second, each substantively reworded item carried a smaller gender gap than the original it replaced (item 5–13, 0.45–0.31; item 9–15, 0.49–0.40; item 12–16, 0.34 to −0.02), so at the item level the EPSI-IT is marginally less gender-differentiating, not more. The gap also tracks internalizing distress: the items with the largest gender gaps are the items most strongly correlated with distress (across items, the two profiles align at *r* = 0.87 in 2025 and 0.95 in 2026; item 3 leads on both), girls reported substantially higher distress (*d* = −0.75 in 2025 and −0.74 in 2026), and adjusting each score for distress reduced the gender gap by about half in 2025 and three-quarters in 2026 (Supplementary Table S11). A cross-lagged panel model showed a reciprocal identity–distress link, with distress preceding clarity somewhat more than the reverse (standardized −0.21 vs. − 0.14).

## 4 Discussion

This study set out to update the measurement of Eriksonian ego identity for contemporary Italian adolescents. We take up first the scale’s structure and scoring, then the validated 11-item EPSI-IT, then the interpretation and limits of the gender-item finding, and finally, 1-year change, gender differences, and the instrument’s practical use.

### 4.1 Structure and scoring

Across both waves, the bifactor model fit best, and the comparison is theoretically informative rather than merely empirical. A strictly unidimensional model is inconsistent with the reliable keying-linked variance the items carry and underfits accordingly; the correlated two-factor model captures that variance but misreads it, promoting a keying method effect to two substantive dimensions; the bifactor representation matches the construct as Erikson framed it, a single bipolar continuum of identity coherence with the synthesis and confusion wordings as method-linked specifics rather than distinct traits (Gnambs et al., 2018; Rodriguez et al., 2016a). The general factor was appreciable in both forms (ω_H_ = 0.62 in the original EPSI, 0.64 in the EPSI-IT; ECV ≈ 0.54), supporting a total identity score and replicating the bifactor finding of Schwartz et al. (2009) and Dimitrova et al. (2019); the two specifics were asymmetric, synthesis retaining reliable variance beyond the general factor (ω_HS_ = 0.57 and 0.65) while clarity did not (0.12 and 0.01), behaving as a method-linked manifestation of general identity. The scoring recommendation follows from the structure rather than from convention: the total score is the primary index, the synthesis subscale a defensible complement, and a clarity subscore, though computable, should not be read as a dimension distinct from general identity.

### 4.2 The revised EPSI-IT

With the gender item removed by design, the revised scale had a cleaner structure, and the bifactor model again fit best. Item review left a single weak item: the candidate replacement (item 14), introduced to adjudicate whether a stability statement indexes synthesis or instead rigidity (Methods), did neither, loading near zero on the general factor with the lowest communality and relating only weakly to self-esteem (*r* = 0.19), with no tie to the distress-laden core of the construct nor to the ideological markers a rigidity reading would predict. Removing it improved hierarchical reliability and yielded the recommended 11-item form. Its measurement held where it matters for use: the harmonized core was scalar-invariant across gender, selection groups, and school track and longitudinally invariant within the panel, and a complementary alignment analysis localized only trivial residual non-invariance to the newest items, so scores are comparable across these groups despite the partially overlapping samples. The nomological network was coherent and theory-consistent, and two features bear interpretation. The moderate inverse relation with aggression was carried by clarity, paralleling the affective-dysregulation signature of distress rather than a weak-externalizing one, which fits an account in which low identity clarity co-occurs with dysregulated affect rather than with rule-breaking. And the gender-polarized tie to gender stereotypes that the original item 8 alone carried left no residue in the EPSI-IT, evidence that removing the item excised construct-irrelevant variance rather than losing signal. The slight 2025-to-2026 attenuation in criterion correlations was reproduced almost exactly on the constant harmonized items (*R*^2^ = 0.96), so it is not specific to the revision; the marginally lower EPSI-IT coefficients are consistent with the cohort advancing rather than with a weaker instrument, though age, period, and residual wording effects are confounded here.

### 4.3 The gender item

One item behaved differently from the rest. The item naming gender had a near-zero general-factor loading and the lowest communality, showed no uniform or non-uniform gender DIF, related near zero to the ideology and values block, and carried the smallest male–female gap of any item, yet had a well-spread distribution and formed a one-item EFA factor. The item that names gender is thus the one that least coheres with the rest of the scale, evidence that it does not operate as an ego-identity indicator. The most parsimonious reading is that it captures content separable from ego identity in this cohort, plausibly subjective certainty about one’s gender identity or role, which the Eriksonian framework folded into ego identity; we neither measured nor validated a gender-identity scale, and the claim rests on the item’s loadings, DIF, and external correlations, not on the single-item EFA factor, consistent with accounts that treat gender identification and gender-role ideology as distinct constructs (Manago et al., 2022). In the terms of contemporary process accounts, in which commitments are formed, explored in depth, and reconsidered across adolescence (Branje et al., 2021; Crocetti et al., 2010), declining to endorse certainty about what being a woman or a man means reads more plausibly as the reconsideration of a commitment than as the absence of one, which is what identity diffusion denotes; an item indexing the former should not load on a general ego-identity factor, and this one does not. A translation artifact is unlikely: the Italian item used the social “donna/uomo” rather than the biological “femmina/maschio” (Methods), the rendering most likely to let it function as a social-identity indicator, and it still did not cohere with ego identity. The weakness was, in retrospect, visible in Schwartz et al. (2009), who listed item 8 among three items failing their variance-explained criterion and remarked that such items “may need to be revised or deleted in future versions of the EPSI” (p. 146) without identifying the reason; the present results supply it, and only the gender item reproduced as anomalous here, while the other two (items 11 and 12) loaded solidly on the general factor (0.58 and 0.58). Response-shift theory rightly warns that apparent item drift can sometimes be true change (Leitgöb et al., 2023); our reading rests on the loading and DIF evidence, not on the temporal comparison alone. The separability is also specific to a general-population sample: in clinical samples, particularly adolescents with marked gender dysphoria, gender-identity uncertainty may reverberate through global identity functioning (the clinical AIDA tradition; Goth et al., 2012; Musetti et al., 2021). What is not defensible is the converse, scoring a generalist instrument so that ordinary exploration of gender lowers measured ego-identity coherence.

### 4.4 One-year change and gender differences

The harmonized common items showed a small 1-year decline in synthesis with stable clarity, replicated within persons. Rather than a fit artifact, this matches the normative U-shaped trajectory in which synthesis dips across early adolescence before rising (Bogaerts et al., 2021), and so speaks to the scale’s sensitivity to developmental change. Boys scored higher than girls in both waves. The gap is unlikely to be a pure measurement artifact, since invariance held through the metric level, the difference persisted under the scalar model and was reproduced under alignment; it cannot, however, be fully separated from the residual intercept-level non-invariance, so it is reported with that caveat. Its substance is largely the internalizing gender difference rather than a deficit specific to identity synthesis: the gap fell almost entirely on the clarity items most saturated with distress (item 3 above all), girls reported substantially higher distress, and adjusting for distress removed about half of the gap in 2025 and three-quarters in 2026 (Supplementary Table S11). Because the confusion items overlap in content with distress, that adjustment partly removes shared construct variance, and the identity–distress link was reciprocal over time with only partial precedence of distress, so the gap is best read as identity coherence and distress being entangled in girls rather than as a pure identity deficit. The gap also narrowed from 2025 to 2026 on the common items, part of the same wave and age effect. Within a grade band, the scale was largely free of nuisance influences, essentially unrelated to age and only weakly to academic achievement. Finally, an exploratory three-item future-orientation factor emerged in EFA but did not survive confirmatory modeling: the items cross-loaded, the factor was unreliable and not separable from synthesis and clarity, and its strongest external tie merely matched the synthesis subscale’s; capturing future orientation would require dedicated item development rather than these items.

### 4.5 Limitations

The strongest caveat is generalizability. The study draws on a single advancing cohort in one Italian metropolitan area, so the reference values are grade-specific (the second year of upper secondary, modal age 15; first-year values are provided separately) and, given a track-stratified design that oversampled male-skewed schools, are reference points rather than population norms, reported with a gender-reweighted total accordingly; independent and culturally diverse samples are needed before the EPSI-IT can be treated as broadly normed. Because the two waves index one cohort, age, period, and cohort are confounded, the 1-year change is within-cohort rather than a secular trend, and the original-EPSI-to-EPSI-IT comparison confounds instrument with wave, which is why inference is anchored to the constant harmonized items. A standalone clarity subscore is not a distinct dimension (ω_HS_ = 0.01), so the dependable scores are the total and the synthesis subscale. The gender-item conclusion is, by construction, about one item in a normative sample; the non-binary subsample was small (*n* = 23 per wave), too small to test whether the item functions differently for non-binary adolescents. Descriptively, the 20 non-binary respondents with valid Wave-1 data gave gender-item responses spanning the full range, with greater dispersion than the binary groups (exact small-cell distributions are kept in the restricted data). This is an open question of particular interest given the item’s content, and one for samples designed to include more gender-diverse youth. The clustered sampling (25 schools) was not modeled, as the working data carry no school identifier; the likely impact is modest (intraclass correlations for student-reported psychosocial outcomes are typically ~0.03; Dong et al., 2016, and invariance held across tracks), bearing mainly on the standard errors of the DIF, latent-mean, and criterion tests, which may be somewhat anti-conservative. Four discriminant criteria are binary, so their point-biserial correlations are conservative, most clearly for the rare hikikomori category. Because the Italian wording was a working adaptation rather than a formally back-translated version (Methods), strict cross-cultural equivalence with the English EPSI is not established; we frame the instrument as an Italian adaptation accordingly, and a bilingual or cross-national design could test EPSI-IT-to-EPSI invariance directly. Finally, all measures are self-report.

### 4.6 Practical implications

For research use, the EPSI-IT offers a short, updated index of Eriksonian identity coherence with concrete scoring guidance: read the total score as the primary index, add the synthesis subscale where a facet is wanted, and do not interpret a clarity subscore as a separate dimension. The grade-specific values are descriptive reference points for research use, not norms: they come from one metropolitan cohort in a single grade band and have not been validated clinically or predictively, so they should not be used to locate an individual adolescent’s standing in applied assessment pending broader norming and independent validation. In general-population Italian adolescent samples of the kind studied here, we recommend omitting the gender item when scoring Eriksonian identity, since retaining it adds construct-irrelevant variance to a measure users read as unchanged, and in applied use can penalize ordinary exploration of gender as though it were identity diffusion. However, whether the item behaves similarly in other cultural and linguistic contexts remains open and can only be settled by independent replication. Those working in other contexts, as well as those whose designs require comparability with historical EPSI data, may prefer to retain the item and report scores computed both with and without it, which would also supply the replication evidence the recommendation needs.

## 5 Conclusion

Ego identity has long been recognized as a construct of considerable explanatory value for youth well-being, yet it is assessed with instruments written decades ago. As the social context in which adolescents form a sense of self continues to change, an identity measure whose items are periodically re-examined against the cohort that answers them, rather than assumed to be stable, is of considerable practical value.

The EPSI-IT is a psychometrically updated, briefer instrument for Eriksonian ego identity in contemporary adolescents. It shows a replicated bifactor structure, with scalar invariance across gender, selection groups, and school track and longitudinal invariance through the loading level on the harmonized core (only a minor intercept-level decrement). Its nomological network is coherent, and its only across-wave attenuation appears equally on the harmonized anchor, which argues against an explanation specific to the revision. It provides preliminary reference values for Italian early adolescents, and illustrates a general point for assessment: a historically situated item, here gender-related certainty used as a marker of ego identity, can come to function as separable content in a contemporary cohort. Continuing to score it as ordinary ego-identity synthesis introduces construct-irrelevant variance into a measure that researchers read as unchanged. That conclusion rests on a single Italian cohort, and independent, culturally diverse samples should now test whether it generalizes.

## Data Availability

The raw data supporting the conclusions of this article will be made available by the authors, without undue reservation.
