# Supplementary material for: Revising the measurement of Eriksonian ego identity in contemporary Italian adolescents: the EPSI-IT
Source: Front Psychol. 2026 Sep 29;17:1927380. doi: 10.3389/fpsyg.2026.1927380 (PMC13658516; doi:10.3389/fpsyg.2026.1927380)
Supplement: Supplementary file 1 [file Data_Sheet_1.PDF]

EPSI-IT: Supplementary Tables

Table E1. Item-by-item mapping of the EPSI to EPSI-IT revision (Open Materials)

#	Subscale	2025 (original, IT)	2026 (revised, IT)	English gloss (2026)	Change	In harmonized set
1	Confusion	Cambio spesso idea su di me	Cambio spesso idea su chi sono	I often change my idea of who I am	light	✓
2	Synthesis	Ho ben chiaro cosa voglio diventare	Ho ben chiaro cosa voglio essere nella vita	I have a clear idea of what I want to be in life	light	✓
3	Confusion	Mi sento confuso/a	Mi sento confuso/a	I feel confused	identical	✓
4	Synthesis	Le cose importanti della vita sono chiare per me	Le cose importanti della vita mi sono chiare	The important things in life are clear to me	light	✓
5 → 13	Synthesis	Ho tutto sotto controllo	Sento di riuscire a gestire quello che mi succede	I feel able to manage what happens to me	**substantive**	—
6	Synthesis	So che tipo di persona sono	So che tipo di persona sono	I know what kind of person I am	identical	✓
7	Confusion	Non riesco a decidere cosa voglio fare della mia vita	Non riesco a decidere cosa voglio fare della mia vita	I can't decide what I want to do with my life	identical	✓
8 → 14	Synthesis	**Ho un forte senso di cosa significhi essere donna/uomo**	Non vedo perché dovrei cambiare	I don't see why I should change	**replaced** (gender item removed)	—
9 → 15	Synthesis	Mi piaccio e sono orgoglioso/a di ciò in cui credo	Mi piace come sono	I like who I am	**substantive**	—
10	Confusion	Non so bene cosa stia facendo in questo periodo	Non so bene cosa stia facendo in questo periodo	I don't really know what I'm doing right now	identical	✓
11	Confusion	Sento di dover indossare una maschera quando sono in compagnia degli altri	Sento di dover indossare una maschera con gli altri	I feel I have to wear a mask with others	light	✓
12 → 16	Confusion	Non mi sento molto coinvolto/a in ciò che faccio	Non penso a costruire il mio futuro	I don't think about building my future	**substantive**	—

Note. Subscale = original EPSI keying (Synthesis = positively keyed; Confusion = reverse-keyed). "In harmonized set" = item retained in the wave-comparable harmonized 8-item form (4 identical + 4 lightly reworded). Items 5, 9, 12 were substantively reworded (new 2026 columns 13, 15,

16); item 8 (gender) was replaced by item 14. The 2026 EPSI-IT comprises items 1,2,3,4,6,7,10,11 + 13,14,15,16; the recommended 11-item form omits item 14. The original English Identity-subscale wordings are not reproduced here and are available in Rosenthal et al. (1981); the original item 8 renders "female/male", which the 2025 Italian adaptation rendered as "donna/uomo" (Methods). The 2025 and 2026 Italian items are the research-team adaptation, and the English entries are working glosses of the administered Italian. *Final-form numbering:* the recommended 11-item EPSI-IT comprises the provenance items 1, 2, 3, 4, 6, 7, 10, 11, 13, 15, 16, renumbered 1–11 for administration (synthesis: provenance items 2, 4, 6, 13, 15; clarity: 1, 3, 7, 10, 11, 16); the provenance numbering is retained throughout this paper to map the revision history.

Table E2. Standardized factor loadings (correlated two-factor model: Synthesis / Clarity)

(A) EPSI original, 2025 (12 items)

Item	Synthesis	Clarity
1		.57
2	.64	
3		.76
4	.66	
5	.73	
6	.73	
7		.62
**8 (gender)**	**.23**	
9	.74	
10		.70
11		.73
12		.72

(B) EPSI-IT, 2026 (11 items)

Item	Synthesis	Clarity
1		.63
2	.70	
3		.75
4	.68	
6	.78	
7		.64
10		.65
11		.62
13	.70	
15	.66	
16		.56

(C) Harmonized 8-item, by wave (common items: 4 unchanged, 4 lightly reworded → Δ best isolates the wave effect)

Item	Factor	λ 2025	λ 2026	Δ (2026 − 2025)
1	Clarity	.58	.65	+.06
2	Synthesis	.75	.77	+.03
3	Clarity	.78	.77	−.01
4	Synthesis	.67	.69	+.02
6	Synthesis	.67	.72	+.06
7	Clarity	.66	.63	−.03
10	Clarity	.69	.65	−.04
11	Clarity	.64	.60	−.04

Note. Standardized loadings from the correlated two-factor model (Synthesis, Clarity; Clarity is the reverse-keyed confusion dimension), ordinal WLSMV, coherence-keyed so that higher scores indicate greater identity coherence; all loadings are positive. Although the bifactor model fit best

overall (Table 1), its orthogonal specific factors were weakly and unstably defined for the EPSI-IT (producing one uninterpretable anomalous specific loading); we therefore report item-level loadings from the two-factor solution, which is sign-consistent and is the model used for the invariance analyses (Table 2). Scoring was verified directly: in the EPSI-IT 11-item form every item's corrected item–total correlation in the coherence direction is positive (.38–.52), confirming correct reverse-keying. (The item-rest values cited in the main text for the common items, e.g. .58/.54, are corrected item–totals within the harmonized 8-item common set, a tighter and more homogeneous base than the 11-item form, which is why they exceed the 11-item ceiling.) The 2025 gender item (8) is a weak synthesis indicator (.23); on the bifactor general factor its loading is −.02. The EPSI-IT replacement item (14) behaves identically (general loading −.07) and is omitted from the recommended 11-item form. **Panel C** reports the harmonized 8-item loadings separately by wave: because the items are common across waves (four unchanged, four lightly reworded), the column Δ best isolates the wave effect, which is small and bidirectional (synthesis loadings if anything rise in 2026, clarity loadings essentially stable, $|\Delta| \leq .06$), indicating that any apparently weaker EPSI-IT (2026) statistics reflect the wave (period and age) and the item revision rather than reduced validity. (Δ computed from unrounded estimates.)

Table E3. Replication of the Study 1 structure across selection groups (original EPSI, 2025, bifactor): leavers (Study 1 only) vs. stayers (panel)

Index	Leavers (*n* = 1,067)	Stayers (*n* = 1,943)
CFI	.935	.929
TLI	.898	.889
RMSEA [90% CI]	.108 [.100, .116]	.103 [.097, .109]
SRMR	.049	.050
ECV (general)	.564	.562
ω_H (general)	.612	.625
Item 8 loading on general factor	−.109	.029
Item 8 communality	.164	.155

Note. The two waves draw largely on different students. To show that the Study 1 findings are not an artifact of the overlap with Study 2, the original-EPSI bifactor is re-estimated in the Study-1-only leavers and, separately, in the panel (stayers; their 2025 responses). The two columns are close throughout, and the gender-item anomaly, a near-zero loading on the general identity factor and the lowest communality in the set, is present in both groups. Stayers *n* = 1,943 (of the 1,963 panel, 20 had incomplete Wave-1 identity data).

Measurement invariance, leavers vs. stayers (two-factor synthesis/clarity, ordinal Wu–Estabrook; adjacent-model Δ):

Level	CFI	RMSEA	SRMR	ΔCFI	ΔRMSEA	ΔSRMR
Configural	.904	.110	.067	—	—	—
Metric	.911	.092	.067	+.007	−.018	+.001
Scalar	.920	.085	.067	+.009	−.008	.000

Full scalar invariance holds across the two groups (CFI rises at each constraint step; RMSEA improves, SRMR flat), so the original-EPSI structure is the same in the Study-1-only leavers and the panel.

Table E4. Replication of the EPSI-IT structure across selection groups (2026, bifactor): entrants (Study 2 only) vs. stayers (panel)

Index	Entrants (*n* = 850)	Stayers (*n* = 1,918)
CFI	.921	.918
TLI	.869	.863
RMSEA [90% CI]	.115 [.105, .125]	.118 [.111, .124]
SRMR	.056	.054
ECV (general)	.477	.566
ω_H (general)	.565	.660
ω_{HS} synthesis	.706	.614
ω_{HS} clarity	.086	.009

Note. The EPSI-IT bifactor is re-estimated in the Study-2-only entrants and, separately, in the panel (stayers; their 2026 responses). Fit is equivalent across groups, and the same asymmetry holds in both: an appreciable general factor, a reliable synthesis specific (ω_{HS} .71 and .61), and a negligible clarity specific (ω_{HS} .09 and .01). General-factor saturation is somewhat lower in entrants (ECV .48, ω_H .57) than in stayers (ECV .57, ω_H .66), but the qualitative structure is the same. Stayers are the same 1,963-respondent panel as in Table E3, here scored in 2026; the stayer *n* is 1,918 (45 excluded for incomplete Wave-2 EPSI-IT data) versus 1,943 in Table E3 (20 excluded in 2025), which is the only reason the two stayer columns differ in *n*.

Measurement invariance, entrants vs. stayers (two-factor synthesis/clarity, ordinal Wu–Estabrook; adjacent-model Δ):

Level	CFI	RMSEA	SRMR	ΔCFI	ΔRMSEA	ΔSRMR
Configural	.888	.119	.069	—	—	—
Metric	.894	.100	.069	+.006	−.020	.000
Scalar	.906	.090	.069	+.012	−.009	.000

Full scalar invariance holds across the two groups, so the EPSI-IT structure is the same in the Study-2-only entrants and the panel.

Table E5. Nomological network across waves: original EPSI (2025) vs. EPSI-IT (2026), full coefficients

Criterion	Scale	*r* 2025 [95% CI]	q_{25}	*r* 2026 [95% CI]	q_{26}	*n* (25/26)
**Well-being / internalizing (convergent)**						
Self-esteem (RSES)	Synthesis	.53 [.50, .55]	<.001	.52 [.49, .55]	<.001	3,010 / 2,768
	Clarity	.60 [.58, .63]	<.001	.53 [.51, .56]	<.001	
	Total	.69 [.67, .70]	<.001	.65 [.62, .67]	<.001	
Future outlook (0–10)	Synthesis	.49 [.46, .51]	<.001	.46 [.43, .49]	<.001	3,010 / 2,768
	Clarity	.39 [.36, .42]	<.001	.38 [.35, .42]	<.001	
	Total	.53 [.50, .55]	<.001	.51 [.49, .54]	<.001	
Life satisfaction (0–10)	Synthesis	.42 [.39, .45]	<.001	.42 [.39, .45]	<.001	3,010 / 2,768
	Clarity	.45 [.42, .48]	<.001	.38 [.35, .41]	<.001	
	Total	.53 [.50, .56]	<.001	.49 [.46, .52]	<.001	
Psychological distress (K10)	Synthesis	−.33 [−.36, −.30]	<.001	−.28 [−.31, −.24]	<.001	3,010 / 2,768
	Clarity	−.57 [−.59, −.55]	<.001	−.49 [−.51, −.46]	<.001	
	Total	−.55 [−.58, −.53]	<.001	−.47 [−.50, −.44]	<.001	
Loneliness (0–10)	Synthesis	−.26 [−.30, −.23]	<.001	−.26 [−.30, −.23]	<.001	3,010 / 2,768

Criterion	Scale	*r* 2025 [95% CI]	q_{25}	*r* 2026 [95% CI]	q_{26}	*n* (25/26)
	Clarity	−.51 [−.54, −.49]	<.001	−.47 [−.49, −.44]	<.001	
	Total	−.48 [−.50, −.45]	<.001	−.45 [−.48, −.42]	<.001	
Alexithymia (PAQ-S)	Synthesis	−.22 [−.25, −.18]	<.001	−.23 [−.27, −.20]	<.001	3,010 / 2,768
	Clarity	−.50 [−.52, −.47]	<.001	−.44 [−.47, −.41]	<.001	
	Total	−.44 [−.47, −.41]	<.001	−.42 [−.45, −.39]	<.001	
Body-image concern	Synthesis	−.26 [−.29, −.22]	<.001	−.26 [−.30, −.23]	<.001	3,010 / 2,768
	Clarity	−.42 [−.45, −.39]	<.001	−.31 [−.34, −.27]	<.001	
	Total	−.41 [−.44, −.38]	<.001	−.35 [−.38, −.32]	<.001	
**Social context**						
Social isolation[b]	Synthesis	−.20 [−.23, −.16]	<.001	−.20 [−.23, −.16]	<.001	3,010 / 2,768
	Clarity	−.35 [−.38, −.32]	<.001	−.30 [−.33, −.26]	<.001	
	Total	−.34 [−.37, −.30]	<.001	−.31 [−.34, −.27]	<.001	
Trust in others†	Synthesis	.25 [.22, .28]	<.001	.25 [.21, .28]	<.001	3,010 / 2,768
	Clarity	.34 [.31, .37]	<.001	.29 [.26, .33]	<.001	
	Total	.36 [.33, .39]	<.001	.33 [.30, .36]	<.001	
School belonging†	Synthesis	.19 [.16, .23]	<.001	.19 [.16, .23]	<.001	3,010 / 2,768
	Clarity	.23 [.20, .26]	<.001	.22 [.18, .25]	<.001	
	Total	.26 [.22, .29]	<.001	.25 [.22, .29]	<.001	
Cyber-victimization	Synthesis	−.16 [−.19, −.12]	<.001	−.16 [−.19, −.12]	<.001	3,010 / 2,768
	Clarity	−.32 [−.35, −.29]	<.001	−.25 [−.29, −.22]	<.001	
	Total	−.30 [−.33, −.26]	<.001	−.25 [−.29, −.22]	<.001	
Bullying victimization	Synthesis	−.16 [−.19, −.12]	<.001	−.14 [−.18, −.11]	<.001	3,010 / 2,768
	Clarity	−.31 [−.34, −.28]	<.001	−.22 [−.25, −.18]	<.001	

Criterion	Scale	*r* 2025 [95% CI]	q_{25}	*r* 2026 [95% CI]	q_{26}	*n* (25/26)
	Total	−.29 [−.32, −.26]	<.001	−.23 [−.26, −.19]	<.001	
Negative school climate	Synthesis	−.07 [−.10, −.03]	<.001	−.14 [−.18, −.10]	<.001	3,009 / 2,768
	Clarity	−.17 [−.20, −.14]	<.001	−.20 [−.24, −.17]	<.001	
	Total	−.15 [−.18, −.11]	<.001	−.21 [−.25, −.18]	<.001	
Hyperconnection[b]	Synthesis	−.12 [−.15, −.08]	<.001	−.06 [−.10, −.02]	.002	3,010 / 2,768
	Clarity	−.23 [−.26, −.19]	<.001	−.14 [−.18, −.10]	<.001	
	Total	−.21 [−.24, −.18]	<.001	−.13 [−.16, −.09]	<.001	
Hikikomori[b]	Synthesis	−.12 [−.15, −.08]	<.001	−.10 [−.14, −.07]	<.001	3,010 / 2,768
	Clarity	−.17 [−.21, −.14]	<.001	−.16 [−.19, −.12]	<.001	
	Total	−.18 [−.21, −.14]	<.001	−.16 [−.20, −.13]	<.001	
**Externalizing / risk**						
Aggression (BPAQ)	Synthesis	−.08 [−.12, −.05]	<.001	−.11 [−.14, −.07]	<.001	3,010 / 2,768
	Clarity	−.46 [−.48, −.43]	<.001	−.40 [−.43, −.37]	<.001	
	Total	−.34 [−.37, −.30]	<.001	−.32 [−.35, −.29]	<.001	
Cyber-perpetration	Synthesis	−.02 [−.06, .02]	.280	−.08 [−.12, −.04]	<.001	3,010 / 2,768
	Clarity	−.13 [−.16, −.09]	<.001	−.15 [−.18, −.11]	<.001	
	Total	−.09 [−.13, −.06]	<.001	−.14 [−.18, −.10]	<.001	
Substance exposure	Synthesis	−.06 [−.10, −.03]	<.001	−.08 [−.11, −.04]	<.001	3,010 / 2,768
	Clarity	−.09 [−.13, −.06]	<.001	−.12 [−.15, −.08]	<.001	
	Total	−.09 [−.13, −.06]	<.001	−.12 [−.16, −.08]	<.001	
Substance tolerance	Synthesis	−.05 [−.08, −.01]	.012	−.07 [−.11, −.03]	<.001	3,009 / 2,767
	Clarity	−.06 [−.10, −.03]	<.001	−.10 [−.14, −.07]	<.001	
	Total	−.07 [−.10, −.03]	<.001	−.11 [−.14, −.07]	<.001	

Criterion	Scale	*r* 2025 [95% CI]	q_{25}	*r* 2026 [95% CI]	q_{26}	*n* (25/26)
Violence tolerance	Synthesis	.00 [−.03, .04]	.951	−.04 [−.07, −.00]	.053	3,008 / 2,767
	Clarity	.02 [−.01, .06]	.208	−.03 [−.07, .01]	.148	
	Total	.02 [−.02, .05]	.405	−.04 [−.08, −.00]	.040	
**Ideology / values (discriminant)**						
Gender stereotypes (GSAS)	Synthesis	.14 [.10, .17]	<.001	.09 [.05, .13]	<.001	3,010 / 2,768
	Clarity	.14 [.10, .17]	<.001	.11 [.07, .15]	<.001	
	Total	.17 [.13, .20]	<.001	.12 [.09, .16]	<.001	
Gender-role attitudes (GRAS)	Synthesis	.12 [.09, .16]	<.001	.05 [.01, .08]	.019	3,010 / 2,768
	Clarity	.14 [.11, .18]	<.001	.10 [.06, .13]	<.001	
	Total	.16 [.13, .19]	<.001	.09 [.05, .13]	<.001	
Gendered traits (GTI)	Synthesis	.02 [−.02, .06]	.287	−.01 [−.05, .03]	.560	3,010 / 2,768
	Clarity	−.06 [−.10, −.03]	<.001	−.03 [−.06, .01]	.183	
	Total	−.03 [−.06, .01]	.128	−.02 [−.06, .01]	.229	
Political orientation	Synthesis	.14 [.11, .18]	<.001	.14 [.10, .18]	<.001	3,010 / 2,767
	Clarity	.12 [.08, .15]	<.001	.13 [.09, .16]	<.001	
	Total	.16 [.12, .19]	<.001	.16 [.13, .20]	<.001	
Religiosity	Synthesis	.09 [.06, .13]	<.001	.11 [.07, .15]	<.001	3,010 / 2,765
	Clarity	.06 [.02, .09]	.003	.07 [.03, .10]	<.001	
	Total	.09 [.05, .12]	<.001	.11 [.07, .14]	<.001	
Meritocratic values[b]	Synthesis	.08 [.04, .11]	<.001	.10 [.06, .14]	<.001	2,921 / 2,565
	Clarity	.09 [.05, .12]	<.001	.10 [.06, .14]	<.001	
	Total	.10 [.06, .14]	<.001	.12 [.08, .16]	<.001	

Note. Pearson r in 2025 (original EPSI) and 2026 (EPSI-IT), each wave scored with its own scale (in 2025 synthesis includes the gender item; subscale composition differs across waves by design; see Table E1). 95% CIs and Benjamini–Hochberg FDR-adjusted q (Benjamini & Hochberg, 1995) are shown for every coefficient; n is the pairwise total-score sample (subscale n equal or marginally larger). After FDR, only some cells of gendered traits (GTI), violence tolerance, and cyber-perpetration are non-significant; given the large N, interpret by magnitude and CI. †Trust-in-others and school-belonging recoded to substantive direction (higher = more). [b] Binary indicator; point-biserial, attenuated by the dichotomous split (most for hikikomori, ≈ 6–7% prevalence). Political orientation coded so higher = more right-wing. Disattenuated r(Synthesis, RSES) = .65 (related but separable). Gender-stratified (2026 total): network largely invariant; only distress (F −.49 vs. M −.38, p = .001) and life satisfaction (F .51 vs. M .42, p = .005) differed. Coefficients are modestly smaller in 2026, a uniform attenuation that Table E6 shows to be a wave (period) effect rather than a property of the EPSI-IT.

Table E6. Nomological network across waves, harmonized 8-item form (common items across waves), full coefficients

Criterion	Scale	*r* 2025 [95% CI]	q_{25}	*r* 2026 [95% CI]	q_{26}	*n* (25/26)
**Well-being / internalizing (convergent)**						
Self-esteem (RSES)	Synthesis	.43 [.40, .45]	<.001	.38 [.35, .41]	<.001	3,010 / 2,768
	Clarity	.59 [.57, .62]	<.001	.54 [.51, .57]	<.001	
	Total	.63 [.61, .65]	<.001	.59 [.57, .61]	<.001	
Future outlook (0–10)	Synthesis	.48 [.45, .50]	<.001	.44 [.41, .47]	<.001	3,010 / 2,768
	Clarity	.39 [.36, .42]	<.001	.36 [.33, .39]	<.001	
	Total	.51 [.48, .54]	<.001	.48 [.45, .51]	<.001	
Life satisfaction (0–10)	Synthesis	.35 [.32, .38]	<.001	.33 [.30, .36]	<.001	3,010 / 2,768
	Clarity	.44 [.41, .47]	<.001	.39 [.36, .42]	<.001	
	Total	.49 [.46, .51]	<.001	.45 [.42, .48]	<.001	
Psychological distress (K10)	Synthesis	−.27 [−.30, −.24]	<.001	−.17 [−.20, −.13]	<.001	3,010 / 2,768
	Clarity	−.57 [−.59, −.54]	<.001	−.51 [−.53, −.48]	<.001	
	Total	−.54 [−.57, −.52]	<.001	−.46 [−.49, −.43]	<.001	
Loneliness (0–10)	Synthesis	−.22 [−.25, −.18]	<.001	−.18 [−.22, −.15]	<.001	3,010 / 2,768
	Clarity	−.50 [−.52, −.47]	<.001	−.48 [−.51, −.45]	<.001	
	Total	−.47 [−.49, −.44]	<.001	−.45 [−.48, −.42]	<.001	
Alexithymia (PAQ-S)	Synthesis	−.17 [−.20, −.13]	<.001	−.16 [−.20, −.12]	<.001	3,010 / 2,768
	Clarity	−.50 [−.52, −.47]	<.001	−.45 [−.48, −.42]	<.001	
	Total	−.44 [−.47, −.41]	<.001	−.42 [−.45, −.39]	<.001	
Body-image concern	Synthesis	−.19 [−.22, −.15]	<.001	−.15 [−.19, −.12]	<.001	3,010 / 2,768
	Clarity	−.41 [−.44, −.38]	<.001	−.33 [−.36, −.29]	<.001	

Criterion	Scale	*r* 2025 [95% CI]	q_{25}	*r* 2026 [95% CI]	q_{26}	*n* (25/26)
	Total	−.39 [−.42, −.36]	<.001	−.32 [−.35, −.29]	<.001	
**Social context**						
Social isolation[b]	Synthesis	−.17 [−.20, −.13]	<.001	−.13 [−.17, −.10]	<.001	3,010 / 2,768
	Clarity	−.35 [−.38, −.32]	<.001	−.30 [−.34, −.27]	<.001	
	Total	−.33 [−.37, −.30]	<.001	−.29 [−.32, −.26]	<.001	
Trust in others†	Synthesis	.20 [.17, .24]	<.001	.20 [.16, .23]	<.001	3,010 / 2,768
	Clarity	.33 [.30, .36]	<.001	.30 [.26, .33]	<.001	
	Total	.34 [.31, .37]	<.001	.32 [.28, .35]	<.001	
School belonging†	Synthesis	.18 [.14, .21]	<.001	.16 [.13, .20]	<.001	3,010 / 2,768
	Clarity	.22 [.18, .25]	<.001	.22 [.18, .25]	<.001	
	Total	.24 [.21, .27]	<.001	.24 [.21, .28]	<.001	
Cyber-victimization	Synthesis	−.12 [−.15, −.08]	<.001	−.12 [−.16, −.08]	<.001	3,010 / 2,768
	Clarity	−.31 [−.34, −.28]	<.001	−.25 [−.29, −.22]	<.001	
	Total	−.28 [−.31, −.25]	<.001	−.25 [−.28, −.21]	<.001	
Bullying victimization	Synthesis	−.13 [−.16, −.09]	<.001	−.09 [−.12, −.05]	<.001	3,010 / 2,768
	Clarity	−.30 [−.33, −.26]	<.001	−.24 [−.27, −.20]	<.001	
	Total	−.28 [−.31, −.24]	<.001	−.22 [−.25, −.18]	<.001	
Negative school climate	Synthesis	−.08 [−.11, −.04]	<.001	−.13 [−.16, −.09]	<.001	3,009 / 2,768
	Clarity	−.16 [−.19, −.12]	<.001	−.20 [−.23, −.16]	<.001	
	Total	−.15 [−.19, −.12]	<.001	−.21 [−.24, −.17]	<.001	
Hyperconnection[b]	Synthesis	−.10 [−.14, −.07]	<.001	−.04 [−.08, −.01]	.022	3,010 / 2,768
	Clarity	−.23 [−.26, −.19]	<.001	−.14 [−.18, −.10]	<.001	
	Total	−.21 [−.25, −.18]	<.001	−.13 [−.16, −.09]	<.001	

Criterion	Scale	*r* 2025 [95% CI]	q_{25}	*r* 2026 [95% CI]	q_{26}	*n* (25/26)
Hikikomori[b]	Synthesis	−.10 [−.13, −.06]	<.001	−.06 [−.10, −.02]	.002	3,010 / 2,768
	Clarity	−.17 [−.20, −.13]	<.001	−.16 [−.20, −.12]	<.001	
	Total	−.17 [−.20, −.13]	<.001	−.15 [−.18, −.11]	<.001	
**Externalizing / risk**						
Aggression (BPAQ)	Synthesis	−.05 [−.09, −.02]	.003	−.06 [−.09, −.02]	.003	3,010 / 2,768
	Clarity	−.45 [−.48, −.42]	<.001	−.41 [−.44, −.37]	<.001	
	Total	−.35 [−.38, −.32]	<.001	−.33 [−.36, −.30]	<.001	
Cyber-perpetration	Synthesis	−.04 [−.07, −.00]	.043	−.09 [−.13, −.05]	<.001	3,010 / 2,768
	Clarity	−.13 [−.16, −.09]	<.001	−.13 [−.16, −.09]	<.001	
	Total	−.11 [−.14, −.07]	<.001	−.14 [−.17, −.10]	<.001	
Substance exposure	Synthesis	−.06 [−.10, −.02]	.001	−.08 [−.12, −.05]	<.001	3,010 / 2,768
	Clarity	−.09 [−.12, −.05]	<.001	−.10 [−.14, −.07]	<.001	
	Total	−.09 [−.13, −.06]	<.001	−.12 [−.15, −.08]	<.001	
Substance tolerance	Synthesis	−.04 [−.07, −.00]	.050	−.06 [−.10, −.02]	.002	3,009 / 2,767
	Clarity	−.06 [−.10, −.02]	.001	−.09 [−.13, −.05]	<.001	
	Total	−.06 [−.10, −.03]	<.001	−.10 [−.13, −.06]	<.001	
Violence tolerance	Synthesis	−.02 [−.06, .02]	.295	−.07 [−.11, −.04]	<.001	3,008 / 2,767
	Clarity	.04 [.00, .07]	.048	.01 [−.03, .05]	.590	
	Total	.02 [−.02, .05]	.340	−.03 [−.07, .01]	.148	
**Ideology / values (discriminant)**						
Gender stereotypes (GSAS)	Synthesis	.12 [.08, .15]	<.001	.04 [−.00, .07]	.056	3,010 / 2,768
	Clarity	.15 [.11, .18]	<.001	.14 [.10, .18]	<.001	
	Total	.16 [.13, .20]	<.001	.12 [.08, .16]	<.001	

Criterion	Scale	r 2025 [95% CI]	q_{25}	r 2026 [95% CI]	q_{26}	n (25/26)
Gender-role attitudes (GRAS)	Synthesis	.09 [.05, .12]	<.001	.00 [−.03, .04]	.828	3,010 / 2,768
	Clarity	.15 [.11, .18]	<.001	.12 [.08, .15]	<.001	
	Total	.15 [.11, .18]	<.001	.09 [.05, .12]	<.001	
Gendered traits (GTI)	Synthesis	.01 [−.03, .04]	.701	−.01 [−.05, .03]	.589	3,010 / 2,768
	Clarity	−.06 [−.10, −.03]	<.001	−.03 [−.06, .01]	.182	
	Total	−.04 [−.08, −.01]	.024	−.02 [−.06, .01]	.206	
Political orientation	Synthesis	.12 [.09, .16]	<.001	.11 [.07, .15]	<.001	3,010 / 2,767
	Clarity	.12 [.09, .16]	<.001	.14 [.10, .18]	<.001	
	Total	.15 [.11, .18]	<.001	.16 [.12, .19]	<.001	
Religiosity	Synthesis	.06 [.03, .10]	<.001	.10 [.07, .14]	<.001	3,010 / 2,765
	Clarity	.06 [.02, .09]	.002	.06 [.02, .10]	.001	
	Total	.07 [.04, .11]	<.001	.10 [.06, .13]	<.001	
Meritocratic values[b]	Synthesis	.08 [.05, .12]	<.001	.11 [.07, .15]	<.001	2,921 / 2,565
	Clarity	.08 [.04, .12]	<.001	.08 [.04, .11]	<.001	
	Total	.10 [.06, .13]	<.001	.11 [.07, .15]	<.001	

Note. Companion to Table E5 on the **harmonized 8-item form** (four items unchanged and four lightly reworded across 2025 and 2026), the best available wave anchor, so any 2025→2026 difference largely reflects a wave (period) effect rather than the scale revision. Regressing the full-scale across-wave attenuation on this common-item attenuation (totals, 26 criteria) gives slope = 1.04, intercept = −.003, $R^2 = .96$, so the attenuation is essentially all a wave effect. 95% CIs and Benjamini–Hochberg FDR q are shown for every coefficient. †Recoded to substantive direction (higher = more). [b] Binary indicator; point-biserial, attenuated by the dichotomous split (most for hikikomori, ≈ 6–7% prevalence).

Table E7. Reference values for the EPSI-IT (Wave 2, second year of upper secondary, modal age 15), item-mean metric (1–5)

Scale	Group	n	M	SD	p5	p10	p25	p50	p75	p90	p95
**Total**	Overall	2,768	3.35	0.73	2.18	2.45	2.91	3.27	3.91	4.36	4.64

Scale	Group	*n*	*M*	*SD*	p5	p10	p25	p50	p75	p90	p95
	Female	1,174	3.18	0.74	2.00	2.27	2.73	3.09	3.64	4.18	4.45
	Male	1,502	3.48	0.69	2.45	2.64	3.00	3.36	4.00	4.45	4.64
	Lyceum	1,139	3.33	0.71	2.18	2.45	2.82	3.27	3.82	4.27	4.55
	Technical	922	3.39	0.74	2.19	2.55	2.91	3.27	3.91	4.45	4.72
	Vocational	634	3.33	0.74	2.27	2.45	2.82	3.18	3.82	4.36	4.64
	Gender-weighted (50/50)	—	3.33	0.73	2.18	2.45	2.82	3.27	3.82	4.36	4.64
**Synthesis**	Overall	2,768	3.30	0.93	1.80	2.00	2.80	3.20	4.00	4.60	5.00
	Female	1,174	3.18	0.89	1.60	2.00	2.60	3.20	3.80	4.40	4.80
	Male	1,502	3.40	0.93	1.80	2.20	2.80	3.40	4.00	4.60	5.00
**Clarity**	Overall	2,768	3.39	0.87	1.83	2.33	2.83	3.33	4.00	4.50	4.83
	Female	1,174	3.18	0.88	1.67	2.00	2.67	3.17	3.83	4.33	4.67
	Male	1,502	3.55	0.83	2.17	2.50	3.00	3.50	4.17	4.67	5.00

Note. Preliminary grade-specific reference values for the **second year of upper secondary** (modal age 15; the wave-2 cohort, predominantly 15-year-olds with a minority of 16), Rome (Roma Capitale); **not population norms**. Boys > girls (gender invariance of the 11-item EPSI-IT held to the loading/metric level, with the boys–girls latent gap persisting under the scalar model; see main text and Table E10). Track differences negligible. Female and Male do not sum to the analytic total: a further 19 respondents reported another gender and 73 had missing gender (gender comparisons contrast Female and Male only); track groups likewise omit cases with missing track. Total and subscales use the same complete-case sample (respondents with all 11 items present), so the three scales share the same *n* within each group. Wave-1 reference values for the original EPSI (first year, modal age 14) are in Table E9.

Table E8. Measurement invariance of the recommended 11-item EPSI-IT (Wave 2), by gender and school track: (A) four-step ordinal sequence and (B) multiple-group alignment

(A) Four-step invariance sequence (ordinal WLSMV, Wu–Estabrook identification)

Grouping	Level	CFI	RMSEA	SRMR	WRMR	ΔCFI	ΔRMSEA	ΔSRMR
**Gender (F vs M)**	Configural	.896	.116	.068	3.40	—	—	—
	Threshold	.888	.108	.068	3.43	−.009	−.008	.000
	Loading	.900	.097	.068	3.46	+.013	−.010	.000
	Intercept	.882	.102	.069	3.97	**−.019**	+.005	+.001
**School track (lyceum/technical/vocational)**	Configural	.886	.121	.071	3.59	—	—	—
	Threshold	.874	.110	.071	3.62	−.012	−.011	.000
	Loading	.890	.098	.072	3.69	+.015	−.012	+.001
	Intercept	.898	.090	.072	3.81	+.009	−.008	.000

Note. Recommended 11-item EPSI-IT (replacement item 14 omitted), Wave 2, two-factor synthesis/clarity model under Wu–Estabrook (2016) ordinal identification (theta parameterization); four-step sequence (configural; equal thresholds; equal thresholds and loadings, the metric level; equal thresholds, loadings, and item intercepts, the scalar level). Δ columns are changes between adjacent nested models. Gender invariance holds through the loading (metric) level, with a small intercept-step decrement (ΔCFI = −.019; ΔRMSEA and ΔSRMR negligible); under the scalar model the boys–girls latent difference persists (synthesis +0.27, clarity +0.48 SD; z = 5.81 and 10.12), so the higher male values are a genuine latent difference rather than a measurement artifact. School-track invariance holds to the scalar level (configural-to-intercept ΔCFI = +.012). WRMR is reported for completeness only (strongly inflated by sample size; DiStefano et al., 2018).

(B) Multiple-group alignment (complementary robustness check)

Factor	Grouping	Groups	R^2 loadings	R^2 intercepts
Synthesis	Gender (F vs M)	2	0.9995	0.9989
Synthesis	School track	3	0.9968	0.9997
Clarity	Gender (F vs M)	2	0.9991	0.9989
Clarity	School track	3	0.9952	0.9998

Factor	Grouping	Group	Aligned mean	Aligned *SD*
Synthesis	Gender (F vs M)	Girls	0.000	1.000
Synthesis	Gender (F vs M)	Boys	0.148	1.092
Synthesis	School track	Lyceum	0.000	1.000

Factor	Grouping		Groups	R^2 loadings	R^2 intercepts
Synthesis	School track	Vocational		−0.007	1.181
Synthesis	School track	Technical		0.054	1.096
Clarity	Gender (F vs M)	Girls		0.000	1.000
Clarity	Gender (F vs M)	Boys		0.672	0.954
Clarity	School track	Lyceum		0.000	1.000
Clarity	School track	Vocational		−0.073	1.061
Clarity	School track	Technical		0.082	1.046

Note. Panel B reports multiple-group alignment (Asparouhov & Muthén, 2014) of the same 11-item form, estimated per factor on group-specific loadings and intercepts with items treated as continuous (sirt 4.2-133; Robitzsch, 2025). R^2 is the proportion of parameter variance consistent with invariance (values near 1 indicate approximate invariance). Aligned means are in SD units with the first group fixed at 0 (girls; lyceum). Largest item-level intercept non-invariance, on the 1–5 metric: item 16 (Clarity, gender, 0.220); item 15 (Synthesis, gender, 0.204); item 7 (Clarity, gender, 0.151); item 2 (Synthesis, track, 0.130); item 7 (Clarity, track, 0.128); all remaining deviations were below 0.13. Alignment is designed for many groups, so with two and three groups these indices are near-ceiling by construction and are reported as corroboration of Panel A, not as an independent test.

Table E9. Reference values for Wave 1, original EPSI (2025, first year, modal age 14), item-mean metric (1–5)

Scale	Group	*n*	*M*	*SD*	p5	p10	p25	p50	p75	p90	p95
**Total**	Overall	3,010	3.34	0.75	2.08	2.33	2.83	3.33	3.92	4.33	4.58
	Female	1,302	3.06	0.73	1.83	2.17	2.58	3.00	3.58	4.08	4.33
	Male	1,688	3.56	0.69	2.42	2.67	3.00	3.50	4.08	4.50	4.67
	Lyceum	1,408	3.31	0.75	2.08	2.33	2.83	3.25	3.83	4.33	4.50
	Technical	1,012	3.41	0.73	2.21	2.50	2.92	3.38	3.92	4.42	4.58
	Vocational	590	3.31	0.78	2.08	2.33	2.75	3.25	3.83	4.42	4.67
	Gender-weighted (50/50)	—	3.31	0.75	2.08	2.33	2.83	3.25	3.83	4.33	4.58
**Synthesis**	Overall	3,010	3.34	0.87	1.83	2.17	2.83	3.33	4.00	4.50	4.67
	Female	1,302	3.12	0.86	1.67	2.00	2.50	3.17	3.67	4.33	4.50
	Male	1,688	3.52	0.84	2.17	2.50	3.00	3.50	4.17	4.67	4.83
**Clarity**	Overall	3,010	3.34	0.95	1.67	2.00	2.67	3.33	4.00	4.67	4.83
	Female	1,302	3.01	0.94	1.50	1.83	2.33	3.00	3.67	4.33	4.67
	Male	1,688	3.60	0.87	2.00	2.50	3.00	3.67	4.33	4.67	4.83

Note. Wave-1 reference values, scored on the **original 12-item EPSI** (synthesis includes the gender item); first year of upper secondary, modal age 14, Rome (Roma Capitale); **not population norms**. Female and Male do not sum to the analytic total (n = 3,010): a further 20 respondents reported another gender (excluded from the Female–Male comparison); Wave 1 had no missing-gender cases. Total and subscales use the same complete-case sample (respondents with all 12 items present), so the three scales share the same n within each group. The boys-higher gap is larger here than in the EPSI-IT (Wave 2, Table E7; see also Table E10).

Table E10. Gender differences by item and wave (Cohen's *d* = boys − girls; coherence-keyed, positive = boys higher)

Scale-level *d*

Score	EPSI original (2025)	EPSI-IT (2026)	Harmonized-8 (2025)	Harmonized-8 (2026)
Synthesis	.47	.25	.37	.08
Clarity	.65	.43	.67	.50
Total	.69	.43	.67	.41

Item-level *d* (— = item not present that wave)

Item	*d* 2025	*d* 2026
1	.57	.38
2	.31	.10
3	**.64**	**.53**
4	.24	.01
5 → 13	.45	.31
6	.31	.09
7	.30	.17
8 (gender)	**.09**	(removed)
9 → 15	.49	.40
10	.36	.30
11	.48	.37
12 → 16	.34	**−.02**

Note. Standardized mean difference (Cohen's *d*), boys − girls, on the coherence-keyed item/scale (positive = boys higher); pooled-*SD* denominator; n = 1,302 girls / 1,688 boys (2025) and 1,174 / 1,502 (2026). Rows 5→13, 9→15, 12→16 pair each substantively reworded item with its replacement (2025 value = original item, 2026 value = its EPSI-IT successor); item 8 (gender) has no 2026 counterpart. The boys-higher gap is present in both waves and is **carried by the clarity items** (item 3 strongest in both, d = .64 / .53; then items 1, 11, 10) far more than by synthesis. The gender-identity item (8) shows the smallest gap of all (d = .09): boys and girls report near-equal certainty about their gender even while they differ on the ordinary identity items, consistent with item 8 tapping a separate dimension. Each reworded item shows a smaller gender gap than the original it replaced (most strikingly item 12 to 16, .34 to −.02). The total gap is larger in 2025 than 2026 and narrows even on the common harmonized items (.67 to .41), so part of the reduction is a wave and age effect rather than the revision.

Table E11. The gender gap in identity coherence and internalizing distress

(A) Gender gap (Cohen's *d*, boys − girls) before and after adjusting for distress (K10)

Form	Score	*d* (raw)	*d* (adjusted for distress)	Reduction
EPSI original (2025)	Item 3 ("confused")	.64	.30	54%
	Clarity	.65	.26	60%
	Total	.69	.32	54%
EPSI-IT (2026)	Item 3 ("confused")	.53	.22	59%
	Clarity	.43	.11	75%
	Total	.43	.11	75%

(B) Cross-lagged panel model, identity ↔ distress (standardized; panel, one-year lag)

Model	Autoregressive identity	Autoregressive distress	Distress → identity	Identity → distress
Harmonized total (n = 1,899)	.48	.63	**−.12**	**−.11**
Clarity (n = 1,900)	.38	.61	**−.21**	**−.14**

Note. Distress = K10. The item most strongly associated with distress is item 3 ("I feel confused/mixed up"), which is also the item with the largest gender gap; across all items, the profile of distress associations and the profile of gender gaps are strongly aligned (r = .87 in 2025, .95 in 2026). Girls report substantially higher distress (d = −0.75 in 2025, −0.74 in 2026). Panel A adjusts each score for distress (residualizing on K10) before recomputing the boys − girls d; roughly half (2025) to three-quarters (2026) of the gender gap is shared with distress. Because the clarity items ("confused", "mixed up") overlap in content with internalizing symptoms, this adjustment partly removes shared construct variance and should be read as a content overlap, not as proof of confounding. Panel B (cross-lagged panel model, MLR; standardized paths, all $p < .001$) shows the identity–distress link is reciprocal; for clarity the distress → identity path (−.21) exceeds the identity → distress path (−.14), indicating partial temporal precedence of distress. A two-wave model on observed composites cannot separate within- from between-person variance; a random-intercept CLPM (≥ 3 waves) is a direction for future work. The clarity model includes one panel member (n = 1,900) who had complete clarity items but a missing non-clarity item, and so was excluded from the total-score model (n = 1,899).

Appendix A. The EPSI-IT (11 items), Italian as administered with English glosses

#	Subscale	Item (Italiano, come somministrato)	English gloss
1	Clarity (R)	Cambio spesso idea su chi sono	I often change my idea of who I am
2	Synthesis	Ho ben chiaro cosa voglio essere nella vita	I have a clear idea of what I want to be in life
3	Clarity (R)	Mi sento confuso/a	I feel confused
4	Synthesis	Le cose importanti della vita mi sono chiare	The important things in life are clear to me
5	Synthesis	So che tipo di persona sono	I know what kind of person I am
6	Clarity (R)	Non riesco a decidere cosa voglio fare della mia vita	I can't decide what I want to do with my life
7	Clarity (R)	Non so bene cosa stia facendo in questo periodo	I don't really know what I'm doing right now
8	Clarity (R)	Sento di dover indossare una maschera con gli altri	I feel I have to wear a mask with others
9	Synthesis	Sento di riuscire a gestire quello che mi succede	I feel able to manage what happens to me
10	Synthesis	Mi piace come sono	I like who I am
11	Clarity (R)	Non penso a costruire il mio futuro	I don't think about building my future

Note. The recommended 11-item EPSI-IT in administration order, rated 1-5 (1 = strongly disagree, 5 = strongly agree). Items marked (R) are reverse-keyed (clarity) and recoded so that higher scores indicate greater identity coherence; synthesis comprises items 2, 4, 5, 9, 10 and clarity items 1, 3, 6, 7, 8, 11. The Italian items are the research team's own adaptation and the English entries are working glosses of the administered Italian, not a back-translated English form (Methods); the original English EPSI Identity items are third-party copyrighted material and are available in Rosenthal et al. (1981). Mapping to the provenance numbering used throughout the paper: 1<-1, 2<-2, 3<-3, 4<-4, 5<-6, 6<-7, 7<-10, 8<-11, 9<-13, 10<-15, 11<-16 (Table E1).
